# Assessing the effectiveness and cost effectiveness of adaptive e-Learning to improve dietary behaviour: protocol for a systematic review

**DOI:** 10.1186/1471-2458-10-200

**Published:** 2010-04-21

**Authors:** Phil Edwards, Lambert Felix, Jody Harris, Elaine Ferguson, Caroline Free, Jane Landon, Karen Lock, Susan Michie, Alec Miners, Elizabeth Murray

**Affiliations:** 1 Department of Epidemiology and Population Health, London School of Hygiene and Tropical Medicine, Keppel Street, London WC1E 7HT, UK; 2 Deputy Chief Executive, National Heart Forum, Tavistock House South, Tavistock Square London WC1H 9LG, UK; 3 Department of Public Health and Policy, London School of Hygiene and Tropical Medicine, Keppel Street, London WC1E 7HT, UK; 4 Research Department of Clinical, Educational & Health Psychology, University College London, 1-19 Torrington Place, London WC1E 7HB, UK; 5 Department of Public Health and Policy, London School of Hygiene and Tropical Medicine, Keppel Street, London WC1E 7HT, UK; 6 Research Department of Primary Care and Population Health, University College London, Holborn Union Building, Archway Campus, Highgate Hill, London N19 5LW UK

## Abstract

**Background:**

The composition of habitual diets is associated with adverse or protective effects on aspects of health. Consequently, UK public health policy strongly advocates dietary change for the improvement of population health and emphasises the importance of individual empowerment to improve health. A new and evolving area in the promotion of dietary behavioural change is e-Learning, the use of interactive electronic media to facilitate teaching and learning on a range of issues, including diet and health. The aims of this systematic review are to determine the effectiveness and cost-effectiveness of adaptive e-Learning for improving dietary behaviours.

**Methods/Design:**

The research will consist of a systematic review and a cost-effectiveness analysis. Studies will be considered for the review if they are randomised controlled trials, involving participants aged 13 or over, which evaluate the effectiveness or efficacy of interactive software programmes for improving dietary behaviour. Primary outcome measures will be those related to dietary behaviours, including estimated intakes of energy, nutrients and dietary fibre, or the estimated number of servings per day of foods or food groups. Secondary outcome measures will be objective clinical measures that are likely to respond to changes in dietary behaviours, such as anthropometry or blood biochemistry. Knowledge, self-efficacy, intention and emotion will be examined as mediators of dietary behaviour change in order to explore potential mechanisms of action. Databases will be searched using a comprehensive four-part search strategy, and the results exported to a bibliographic database. Two review authors will independently screen results to identify potentially eligible studies, and will independently extract data from included studies, with any discrepancies at each stage settled by a third author. Standardised forms and criteria will be used.

A descriptive analysis of included studies will describe study design, participants, the intervention, and outcomes. Statistical analyses appropriate to the data extracted, and an economic evaluation using a cost-utility analysis, will be undertaken if sufficient data exist, and effective components of successful interventions will be investigated.

**Discussion:**

This review aims to provide comprehensive evidence of the effectiveness and cost-effectiveness of adaptive e-Learning interventions for dietary behaviour change, and explore potential psychological mechanisms of action and the effective components of effective interventions. This can inform policy makers and healthcare commissioners in deciding whether e-Learning should be part of a comprehensive response to the improvement of dietary behaviour for health, and if so which components should be present for interventions to be effective.

## Background

### The need for improved dietary behaviour

The composition of habitual diets is associated with adverse or protective effects on health [1-3]. Specifically, diets high in saturated fats and sodium have been found to increase risk of cardiovascular diseases, while those high in fruit and vegetables and low in saturated fats have been linked with reductions in a range of diseases including certain cancers, cardiovascular disease and hypertension [4-7]. The WHO reports that the consumption of up to 600 g per day of fruit and vegetables could reduce the total worldwide burden of disease by 1.8%, and reduce the burden of ischaemic heart disease and ischaemic stroke by 31% and 19% respectively [8]. In the UK, the consumption of fruits and vegetables, dietary fibre, iron (pre-menopausal women only) and calcium are well below recommendations, whereas intakes of saturated fats and sodium exceed recommendations in large sections of the population [9]. Consequently, UK public health policy strongly advocates dietary change for the improvement of population health and emphasises the importance of individual empowerment to improve health [7,10], thereby shifting the focus of the National Health Service from treatment to prevention of illness [11,12].

### Adaptive e-Learning via interactive computerised interventions

**Figure 1:**
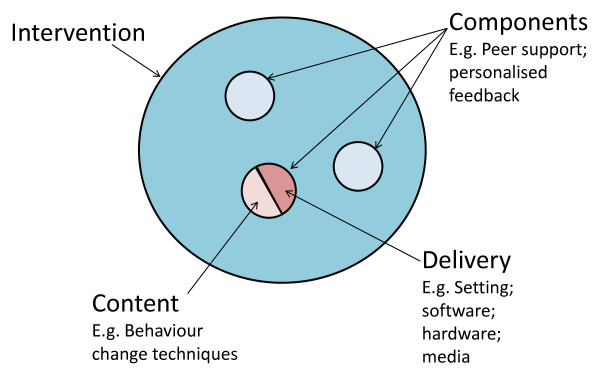
**Conceptual framework of an intervention**.

**Figure 2:**
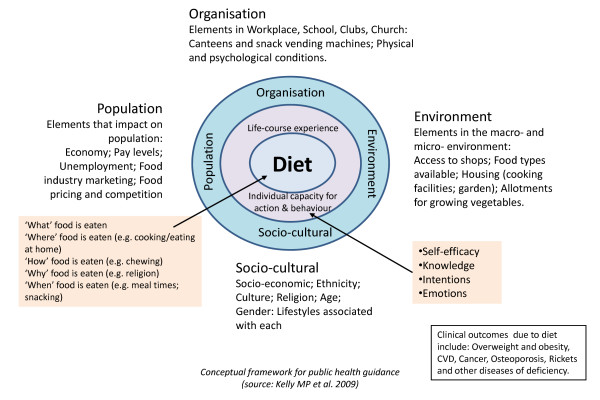
**Conceptual framework for explanations of behaviour change**. Source: Adapted from [43]

A new and evolving area in the promotion of dietary behavioural change is e-Learning, the use of interactive electronic media to facilitate teaching and learning on a range of issues including health (see Additional file 1 for definitions of terms used in e-Learning). E-Learning has grown out of recent developments in information and communication technology, such as the Internet, interactive computer programs, interactive television, and mobile phones [13-17], technologies which are fast becoming more accessible to the general population. (For example, an estimated 70% of the population in the UK has access to the Internet and this percentage is likely to continue to grow [18].) This high level of accessibility with emerging advances in computer processing power, data transmission and data storage makes interactive e-Learning a potentially powerful and cost-effective medium for improving dietary behaviour [19-21]. It also has a number of distinct advantages compared with traditional approaches for the promotion of dietary behaviour change, such as the possibility of tailoring to individual circumstances [22], translating complex information through video, graphics, and audio systems [23], and potential cost savings on face-to-face interventions involving healthcare practitioners. The evidence that individualised, tailored e-Learning approaches are more effective than traditional non-tailored interventions [24] has given them a promising lead in health education [25-27]. E-Learning interventions have been classified into three generations: 1^st ^generation interventions use computers to tailor print materials; 2^nd ^generation interventions use interactive technology delivered on computers; and 3^rd ^generation interventions use portable devices such as mobile phones, for more immediate interaction and feedback [28]. Exploration of the properties of different e-Learning interventions is now required in order to determine possible effective components (with each component comprising both delivery and content- see fig 1). Potential cognitive and emotional mediators of dietary behaviour change should also be explored, in order to elicit potential mechanisms of action (see fig 2).

There is a risk that e-Health and use of new technologies in health care might widen health inequalities on either side of the 'digital divide'. Experience suggests that there are two dimensions to the digital divide and its impact on health inequalities: *access *(to physical hardware and software) and *accessibility *(or the ability of people with differing literacy/health literacy/IT literacy to use or apply information and support supplied through e-Learning). It has been shown that it is possible to deliver e- health interventions specifically designed for people with low literacy skills (e.g. Hispanics in Southern USA, [29], homeless drug users [30], and single teenage mothers [31]). What remains less clear is the extent to which people with low literacy skills will feel comfortable using a computer, or will be able to act on information or advice provided over the Internet.

Interactive e-Leaning programmes to promote positive dietary behavioural changes have the potential to benefit population health. However, before e-Learning can be hailed as a dietary behaviour change intervention of the future, the effective components and mechanisms of action of e-Learning programmes must be identified, and its cost-effectiveness established in different contexts.

### Previous reviews

Three systematic reviews have examined the effectiveness of e-Learning for dietary behaviour change. The first [32] was restricted to first-generation interventions for dietary change and did not include any web or Internet-based interventions. The second [33] examined a broad range of second-generation interactive interventions for dietary behaviour change. Both of these reviews reported studies published prior to 2006 that were carried out in a variety of settings. The third review [28] was more recent, reviewing second- and third-generation interventions trialled up to 2008, but only in primary prevention in adults (no participants with diagnosed disease). All reviews were restricted to publications in the English language, and limited their searches to relatively few databases, increasing the potential for publication bias. The conclusions drawn from these systematic reviews were that e-learning shows some promise for dietary behaviour change, although the findings were mixed. Inter-study heterogeneity with respect to study design, participants, measures, and outcomes precluded meta-analysis to estimate pooled intervention effects. Moreover, the cost-effectiveness of e-Learning was not evaluated in any review, nor was there any attempt to identify potential mechanisms of action. The third review assessed internal and external validity of trials, and began to isolate effective components.

Our review will provide a comprehensive and up-to-date account of e-Learning technologies in use for promoting dietary behavioural change, and an evaluation of their effectiveness and cost-effectiveness in improving dietary behaviour as well as clinical outcomes. We will investigate the psychological theories that underlie the process of behaviour change [34-36], and look for key behaviour-change techniques that have been shown to be associated with healthy eating behaviours [37]. Where these have been used to inform intervention design in trials, we will explore potential mediators of behaviour such as knowledge, intention, self-efficacy and emotions with a view to understanding mechanisms of action. We will also explore the different components of trialled interventions, in order to find the effective components of successful e-Learning interventions for dietary change.

We will use a systematic search strategy (described below) to identify relevant studies and to reduce the potential for reporting biases, and use wider inclusion criteria than in previous reviews to enable a wider range of conclusions to be drawn. Preliminary literature searching, including the NHS's Economic Evaluation Database, suggests that the published evidence on cost-effectiveness is extremely limited. Therefore, we will conduct a de novo economic evaluation of the intervention studies, looking at cost-effectiveness in England and Wales, if the required clinical effectiveness data are available from the primary trials. We will conclude with policy recommendations and recommendations for future primary research.

### AIMS of the Review

The aims of this systematic review are to determine the effectiveness and cost-effectiveness of adaptive e-Learning for improving dietary behaviours. The specific objectives are to:

• Describe the range of e-Learning technologies in use for promoting dietary behavioural change;

• Evaluate interactive e-Learning effectiveness in terms of improvement in dietary behaviour and clinical outcomes;

• Explore the properties of different e-Learning interventions in order to determine possible effective components of successful e-Learning interventions for dietary behaviour change;

• Investigate potential explanations of dietary behaviour change, and mechanisms of action;

• Evaluate cost-effectiveness compared with current standard interventions, and likely budget impact in England & Wales.

Final outputs will be a report to the UK National Institutes of Health Research (NIHR) Health Technology Assessment (HTA) programme, and a peer-reviewed paper.

## Methods/Design

### Design

The research will consist of a systematic review and a cost-effectiveness analysis.

### Criteria for considering studies

#### Types of study

We will include randomised controlled trials (RCTs) for evidence of effectiveness, and economic evaluations for evidence of cost-effectiveness.

#### Types of population

Adolescents or adults aged 13 years and above who have participated in a study designed to evaluate the effectiveness of e-Learning to promote dietary behavioural change. We shall include all clinical conditions where dietary advice plays a major part in case management.

#### Types of intervention

Interventions will be included if they are interactive computer software programmes that tailor output according to user input (second and third generation interventions). These include those where users enter personal data or make choices about information that alter pathways within programmes to produce tailored material and feedback that is personally relevant. Users may interact with the programmes as members of a small group, as well as individually. Programmes should be available directly to users and allow independent access without the need for any expert facilitation.

Interventions will be excluded if they are: First-generation tailored 'information only' (e.g. providing a leaflet or PDF); simple information packages with no interactive elements; non-interactive mass media interventions (such as TV advertisements); interventions designed to be used with others' help (e.g. teacher or health professional); interventions targeted at health professionals or teachers; computer-mediated delivery of individual health-care advice (e.g. online physicians); or electronic history-taking or risk assessment with no health promotion or interactive elements.

#### Outcome measures

We anticipate that most interventions will be aimed at dietary behaviours, and are unlikely to have followed participants long enough to obtain clinical changes. However, as dietary behaviour tends to be self-reported it is prone to error (e.g. recall bias). Biological outcomes on the other hand are more objective and also more important for modelling purposes. We will therefore use dietary behaviour as our primary outcome, but we will attempt to obtain data that allow us to model the relationship between behaviours and clinical changes.

#### Primary outcome measures

The primary outcome variables will be those related to dietary behaviours. They will include estimated intakes or changes in intake of energy, nutrients, dietary fibre, foods or food groups. The dietary assessment tools or techniques used to estimate dietary behaviour will be critically examined in terms of quality.

#### Secondary outcome measures

Objective measures that are likely to respond to changes in dietary behaviours and are associated with adverse clinical outcomes will be examined, including measurements of anthropometric status and blood biochemistry.

#### Other data

We will also seek data on economic outcomes, including costs of providing the intervention and costs to the individual user; data on unintended adverse consequences of the interventions; and process outcomes (e.g. usage data). Data relating to potential cognitive and emotional mediators of dietary behaviour will also be extracted. These will only be extracted if primary and/or secondary outcome data are available.

### Identification of eligible studies and data extraction

#### Search strategy

We have designed a four-part search strategy: Firstly, we will search electronic bibliographic databases for published work (see below for databases to be searched). Secondly, we will search the grey literature for unpublished work. Thirdly, we will search trials registers for ongoing and recently completed trials. Finally, we will search reference lists of published studies and contact authors and e-health research groups to check for more trials. All databases will be searched from 1990 (any studies conducted in the 1980s will be identified by searching the reference lists of included studies). There will be no restrictions by language. To ensure the review is reasonably up-to-date at reporting, the searches will be re-run immediately prior to analysis and further studies retrieved for inclusion. The search strategy comprises two concepts: Computer/Internet-based interventions, and dietary behaviour. (See Additional file 2 for full search strategies).

The databases that will be searched are:

CINAHL, Cochrane Library, Dissertation Abstracts, EMBASE, ERIC, Global Health, HEED, HMIC, MEDLINE, PsychInfo, and Web of Science.

#### Screening and review process

All studies identified through the search process will be exported to a bibliographic database (EndNote version X3) for de-duplication and screening. Two review authors will independently examine the titles, abstracts, and keywords of electronic records for eligibility according to the inclusion criteria above. Results of this initial screening will be cross-referenced between the two review authors, and full-texts obtained for all potentially relevant reports of trials. Full-texts of potentially eligible trials will go through a secondary screening by each reviewer using a screening form based on the inclusion criteria (see Additional file 3) for final inclusion in the review, with disagreements resolved by discussion with a third author. Reference lists of all eligible trials will be searched for further eligible trials.

#### Data extraction

Two review authors will independently extract relevant data using a standardised data extraction form (Additional file 4) in conjunction with a data extraction manual (Additional file 5). Trial managers will be contacted directly if the required data are not reported in the published study.

### Analysis

#### Descriptive analysis

We will describe all studies that meet the inclusion criteria, including:

1. Study design

a. Trial design and quality

b. Data collection methods, modes, and techniques; validity of tools

c. Adherence to protocol (We will attempt to retrieve the protocols of eligible studies to examine the adherence to initial plans).

d. Statistical and other analyses

e. Conflict of interest

2. Participants (intervention and control)

a. Socio-economic and demographic characteristics (e.g. age, ethnicity, education level)

b. Health status: diagnosed disease (e.g. diabetes, cardiovascular disease, obesity) vs. no diagnosed disease

c. Technological literacy and access to technology

d. Psychological characteristics (e.g. help seeking)

3. Intervention

a. Setting and recruitment methods

b. Components of the intervention, including delivery and content

c. Frequency, intensity and duration of the intervention

d. Behaviour change theories employed in intervention design

4. Outcomes

a. Primary and secondary outcomes measured

b. Information on process (ease of use) and usage (compliance)

Information on how access to the intervention was provided (e.g. free laptops/Internet access); the intended reading age (or other measure of technological literacy/skill required); and the socio-demographic characteristics of the participants will be used to address concerns over the digital divide. Where primary studies have included sub-group analyses of users with low-income or low educational status, we will note these. If sufficient data are provided by the primary studies we will consider undertaking sub-group analyses of intervention effects in low-income and low educational status users.

#### Statistical analysis

We will use statistical software (Stata version 11) for data synthesis. In the presence of sufficient homogeneity (i.e. comparable population, interventions and outcomes) we will pool the results of RCTs using a random-effects model, with standardised mean differences (SMDs) for continuous outcomes and odd ratios for binary outcomes, and calculate 95% confidence intervals and two sided P values for each outcome. In studies where the effects of clustering have not been taken into account, we will adjust the standard deviations by the design effect, using intra-class coefficients if given in papers, or using external estimates obtained from similar studies [38]. In the absence of sufficient homogeneity, we will present tables of the quantitative results.

We will assess selection bias using Egger's weighted regression method and Begg's rank correlation test. Heterogeneity among the trials' odds ratios will be assessed by using both χ^2 ^test at a 5% significance level and the *I*^2 ^statistic, the percentage of among-study variability that is due to true differences between studies (heterogeneity) rather than to sampling error. We will consider an *I*^2 ^value greater than 50% to reflect substantial heterogeneity. We will conduct sensitivity analyses in order to investigate possible sources of heterogeneity including study quality (adequate vs. inadequate allocation concealment; low vs. high attrition) and socio-demographic factors that could act as effect modifiers (for example age, gender, sexuality and socioeconomic status). Details of each e-Learning programme will be presented in a table of study characteristics, and we will conduct exploratory, descriptive analyses of data available on effective components and mechanisms of action.

#### Economic evaluation

A decision-analytic model will be built to assess cost-effectiveness, so that intervention effects identified by the systematic review can be extrapolated beyond the observed trial periods [39]. The aim of the evaluation will be to compare the cost-effectiveness of adaptive e-learning technologies against other dietary interventions available in England and Wales. We will combine the results of the systematic review with expert advice to identify the relevant e-learning technologies and appropriate comparators (e.g. group learning, individual contact with a dietician) and model the costs associated with each.

The primary form of economic evaluation will be a cost-utility analysis, where health outcomes are expressed as quality-adjusted life-years (QALYs). The base case analysis will be performed from a NHS cost perspective. Future costs and health benefits will be discounted at 3.5% per annum. Results will be presented as expected costs, expected QALYs, incremental cost-effectiveness ratios, net benefit statistics and cost-effectiveness acceptability curves.

The model structure will be informed by: (i) reviewing previously published decision models where the immediate objective has been to evaluate technologies designed to help people change dietary behaviour and (ii) the results of the systematic review with respect to the recorded outcomes. For example, if the trials report changes in BMI, then a Markov model could be constructed, with the health states defined in terms of BMI groupings. Intervention costs [40] and effects could then be simulated by movements through these health states, with higher BMI being associated with increased health care costs (including costs of health outcomes such as cardiovascular disease, type 2 diabetes and cancer) and increased probabilities of all-cause mortality from sources such as the British Regional Heart Study [41].

Depending on the chosen model structure, other literature reviews will also be performed to identify evidence for other parameters, such as the increased costs and the dis-utility associated with increasing levels of obesity. Other variables for which additional searches might be required could include evidence linking increases in fruit or vegetable intake with weight loss and the reduction in the likelihood of cardiovascular disease following weight loss. Other important issues to incorporate in the model structure are likely to include attrition from the intervention, non-compliance and the need to retain a degree of flexibility as clinical studies are likely to report different outcomes (e.g. changes in behavioural and clinical outcomes).

If the primary systematic review identifies a 'network' of relevant RCTs, consideration will be given to performing formal mixed- or indirect-treatment comparisons to allow cost-effectiveness comparisons to be made across all programmes [42].

#### Stakeholder involvement

Involvement of non- governmental organisations who represent a range of potential user groups has been an important part of the project development. Jane Landon, Deputy Chief Executive of the National Heart Forum, is a member of the investigative team, attends steering group meetings with the other co-investigators, and contributes to decisions made as the study progresses. The National Heart Forum (NHF)is an alliance of over 60 national organisations representing professional, academic, consumer, charity and public sector organisations throughout the UK, and therefore represents a large population of potential users of e-Learning for dietary behaviour change. In our experience, user input is particularly valuable in considering outcomes of interest to users, and methods of disseminating results to user communities, thus contributing to public involvement in science.

## Discussion

### Strengths and limitations of the review

Strengths of this review include unambiguous definitions and inclusion criteria, and a clear and systematic approach to searching, screening and reviewing studies and extracting data using standardised forms and duplicating all stages. Our search area is large enough and our inclusion criteria broad enough to encompass the broadest range of interactive e-Learning interventions and dietary, clinical and behavioural outcomes, and so has the best chance of identifying effective components of effective interventions for translation into policy or further research. Our review will also pinpoint potential mechanisms of action in terms of psychological theories of behaviour change employed in interventions, which will further inform the future development of e-learning interventions. The final report to the HTA will allow for a comprehensive statistical, economic and subgroup analyses, as well as descriptive analysis not usually available given the limited space available in academic journals.

Although every effort will be made to locate unpublished trials our findings may still be vulnerable to selective reporting, and despite a pre-defined and systematic approach to screening and reviewing the study will still involve judgments made by review authors, either of which may lead to bias. This review will not look at cohort or other observational study designs, and therefore may not be able to evaluate acceptability or preference of e-Learning interventions.

### Implications for policy and healthcare commissioning

This review aims to provide comprehensive evidence of the effectiveness and cost-effectiveness of adaptive e-Learning interventions for dietary behaviour change, and explore potential psychological mechanisms of action and the effective components of effective interventions. This can inform policy makers and healthcare commissioners in deciding whether e-Learning should be part of a comprehensive response to the improvement of dietary behaviour for health, and if so which components should be present for interventions to be effective.

## Competing interests

The authors declare that they have no competing interests.

## Authors' contributions

PE will manage the project, and provided expertise in the design of the systematic review and statistical analysis; LF and JH will undertake screening, reviewing, and data extraction, and produced the various tools to be used in the review; LF will also undertake all database searching; JH will also provide nutrition expertise and coordinate writing and publication; EF provided expertise and guidance regarding nutritional outcomes to be investigated; CF, EM, SM and KL contributed materially to the design of the conceptual framework and the systematic review methods; SM also provided expertise and guidance regarding behavioural theories to be investigated; AM designed and will undertake the cost-effectiveness analysis; JL represented user groups in the design of the review. All authors contributed to the writing or editing of the protocol for publication, and will contribute to the final report and paper.

## Pre-publication history

The pre-publication history for this paper can be accessed here:

http://www.biomedcentral.com/1471-2458/10/200/prepub

## Supplementary Material

Additional file 1 Glossary. Click here for file

Additional file 2 **Search strategies (Word file).** Search strategies to be run in Medline database to identify potentially eligible studies for the review. (The Medline strategy is indicative of strategies to be run in all specified databases). Click here for file

Additional file 3 **Screening form (Word file).** The form used to identify eligible studies. Click here for file

Additional file 4 **Data extraction form (Word file).** The form used to extract data from eligible studies. Click here for file

Additional file 5 **Data extraction manual (Word file).** A manual to guide data extraction. Click here for file
