# Supplementary material for: Assessing the effectiveness and cost effectiveness of adaptive e-Learning to improve dietary behaviour: protocol for a systematic review
Source: BMC Public Health. 2010 Apr 21;10:200. doi: 10.1186/1471-2458-10-200 (PMC2868000; doi:10.1186/1471-2458-10-200)
Supplement: Additional file 1 — Glossary. [file 1471-2458-10-200-S1.DOC]

**Box 1: Glossary**

| *Adaptive*............. | Requires contributions from users (e.g. entering personal data, making choices) which alter pathways within programmes to produce tailored material and feedback that is personally relevant to users of the programme [1]. |
| --- | --- |
| *Diet*..................... | Food that is eaten (may be categorised into food groups), including fortified or functional foods but excluding supplements. |
| *Dietary behaviour*............ | Food intake (including what, where, how, and when food is eaten). |
| *e..........................* | Anything electronic (e.g. SMS, digital TV, PDA, CD-ROM, Internet, etc.) |
| *Learning*.............. | Where the goal of the intervention was to improve dietary knowledge and / or behaviour. |
| *Mediator.............* | A variable that describes how effects will occur, by accounting for the relationship between the independent and dependent variables. |
