# Supplementary material for: Assessing the effectiveness and cost effectiveness of adaptive e-Learning to improve dietary behaviour: protocol for a systematic review
Source: BMC Public Health. 2010 Apr 21;10:200. doi: 10.1186/1471-2458-10-200 (PMC2868000; doi:10.1186/1471-2458-10-200)
Supplement: Additional file 2 — Search strategies (Word file). Search strategies to be run in Medline database to identify potentially eligible studies for the review. (The Medline strategy is indicative of strategies to be run in all specified databases). [file 1471-2458-10-200-S2.DOC]

**MEDLINE**

**DIETARY (A)**

1. exp "food and beverages"/
2. exp Diet/
3. exp nutrition processes/ or exp nutritional requirements/ or exp nutritional status/ or exp nutritive value/
4. exp Nutrition Therapy/
5. exp nutrition assessment/
6. exp Body Weight/
7. exp Nutrition Disorders/
8. food$.ab,ti.
9. nutri$.ab,ti.
10. diet$.ab,ti.
11. weigh$.ab,ti.
12. (diet$ adj3 behav$).ab,ti.
13. (eat$ adj3 behav$).ab,ti.
14. 1 or 2 or 3 or 4 or 5 or 6 or 7 or 8 or 9 or 10 or 12 or 12 or 13 **(A)**

**E (B)**

1. exp Electronics/
2. exp cybernetics/ or exp reminder systems/ or exp communications media/ or exp computing methodologies/ or computers/ or exp informatics/
3. exp Audiovisual Aids/
4. exp Technology/
5. exp decision support techniques/
6. online.ab,ti.
7. computer$.ab,ti.
8. internet.ab,ti.
9. (World wide web or world-wide-web or world-wide web or website$ or internet$).ab,ti.
10. (chat room$ or chatroom$).ab,ti.
11. (email or e-mail or electronic messag$).ab,ti.
12. (blog$ or web-blog$ or weblog$).ab,ti.
13. (bulletin board$ or bulletinboard$ or message board$ or message board$).ab,ti.
14. (DVD or dvd).ab,ti.
15. (CD-ROM or cd-rom or CDROM or cdrom).ab,ti.
16. interactive health communicat$.ab,ti.
17. interactive televis$.ab,ti.
18. interactive video$.ab,ti.
19. interactive technolog$.ab,ti.
20. interactive multimedia.ab,ti.
21. (E-health or ehealth or electronic health).ab,ti.
22. (surf or surf$ or browse or brows$).ab,ti.
23. (iphone or i-phone).ab,ti.
24. (ipod or i-pod).ab,ti.
25. (information kiosks or inform$ kiosk$).ab,ti.
26. (short messaging service or sms or text message or text$ message or txt).ab,ti.
27. (multimedia messaging service or mms).ab,ti.
28. virtual reality.ab,ti.
29. 15 or 16 or 17or 18 or 19 or 20 or 21 or 22 or 23 or 24 or 25 or 26 or 27 or 28 or 29 or 30 or 31 or 32 or 33 or 34 or 35 or 36 or 37or 38 or 39 or 40 or 41 or 42 **(B)

     STUDY DESIGN (C)**
30. Randomi#ed controlled trial.pt.
31. controlled clinical trial.pt.
32. Randomi#ed.ab.
33. placebo.ab.
34. clinical trials as topic.sh.
35. randomly.ab.
36. trial.ti.
37. 44 or 45 or 46 or 47 or 48 or 49 or 50
38. (animals not (humans and animals)).sh,ti.
39. 51 not 52 **(C)

     PUBLICATION YEAR (D)**
40. (199$ or 200$).yr. **(D)

    A+B+C+D**
41. 14 (A) + 43(B) + 53 (C) + 54 (D)
