# Supplementary material for: Assessing the effectiveness and cost effectiveness of adaptive e-Learning to improve dietary behaviour: protocol for a systematic review
Source: BMC Public Health. 2010 Apr 21;10:200. doi: 10.1186/1471-2458-10-200 (PMC2868000; doi:10.1186/1471-2458-10-200)
Supplement: Additional file 3 — Screening form (Word file). The form used to identify eligible studies. [file 1471-2458-10-200-S3.DOC]

1. **Design**

| Is this a randomised controlled trial, evaluating the effectiveness of an intervention? | No | Yes | ? |
| --- | --- | --- | --- |
|  | Go to 5 | Go to 2 | Go to 2 |

*Note: Exclude (but read for background and check reference list) any systematic or non-systematic reviews of interventions, and any non-RCT evaluations of e-Learning interventions.*

1. **Participants**

| Are participants adults or adolescents aged 13 years and above? | No | Yes | ? |
| --- | --- | --- | --- |
|  | Go to 5 | Go to 3 | Go to 3 |

*Note: Interventions may be targeted to populations as a preventative measure, or to populations with clinical conditions for management of these conditions. Interventions may be targeted to individuals or their carers.*

1. **Intervention(s)**

| Does the intervention seek to change behaviour through interactive software programmes, delivered through electronic media, which tailor output according to user input? | No | Yes | ? |
| --- | --- | --- | --- |
|  | Go to 5 | Go to 4 | Go to 4 |

*Note: Users may interact with the programme as members of a group or as individuals, but may not require expert facilitation. Multi-component interventions including other outcomes (eg. physical activity) will be included only if the dietary component can be isolated.*

1. **Outcomes**

| Does the study include any of the following as outcomes: Dietary behaviour; food consumption; energy intake; nutrient or dietary fibre consumption; BMI; blood lipid levels; plasma vitamin or mineral levels or biomarkers of these; or a combination of these outcomes? | No | Yes | ? |
| --- | --- | --- | --- |
|  | Go to 5 | Go to 5 | Go to 5 |

1. **Decisions**

| If all 1–4 ‘Yes’ | Paper to be included. Data extraction form to be completed. Paper and form to be filed on J drive. |
| --- | --- |
| If any 1–4 ‘No’ | Paper to be excluded. Reasons for exclusion to be entered on ‘exclusion’ database. Paper to be filed on J drive. |
| If any 1–4 ‘?’ | Reviewer 1 and 2 (and if necessary 3) to reach consensus;  include / exclude as above. |
| Possible background papers | Reference lists to be checked. Paper to be filed on J drive for reference. |

1. **References**

| Reference list checked? | Yes | No |
| --- | --- | --- |
