# Supplementary material for: Assessing the effectiveness and cost effectiveness of adaptive e-Learning to improve dietary behaviour: protocol for a systematic review
Source: BMC Public Health. 2010 Apr 21;10:200. doi: 10.1186/1471-2458-10-200 (PMC2868000; doi:10.1186/1471-2458-10-200)
Supplement: Additional file 4 — Data extraction form (Word file). The form used to extract data from eligible studies. [file 1471-2458-10-200-S4.DOC]

**Review author**:

**Date**:

**Author**:

**Year of publication**:

**Country**:

**Title**:

|  |
| --- |

**Aims & Objectives**:

|  |
| --- |

1. **METHODS:**
2. **Recruitment**
   Electronic Mail shot Telephone Advert in clinic Advert elsewhere Social media networks

Word of mouth Physician / health professional referral  Organisation referral

Other (specify):

1. **Study Design**
   Parallel-group trial Crossover trial Cluster RCT Factorial trial
2. **Randomisation *-* Sequence generation**

Simple randomisation, tossing a coin
 Simple randomisation, random number table

Simple randomisation, computerised random number generator
Random permuted blocks

Minimisation

Response-adaptive designs
Randomised consent design (Zelen design)

Stratified randomisation

Other (specify):

Not specified

1. **R****andomization - Allocation concealment**

Centralised randomisation by telephone – automated

Centralised randomisation by telephone – person
Web-based randomisation

Sequentially numbered, sealed envelopes

Sealed envelopes not sequentially numbered or not opaque
Random numbers read by someone not entering patients into trial (closed list)
Random numbers read by someone entering patients into trial (open list)
Other (specify):

Not specified

1. **Randomisation – Implementation**
2. Who **generated** the allocation concealment? Known Unknown
   If Known, then specify:
3. Who **enrolled** participants? Known Unknown
   If Known, then specify:
4. Who **assigned** participants to their groups? Known Unknown
   If Known, then specify:
5. **Analysis**
    Intention to treat analysis Per protocol analysis  Not specified
6. **Times at which outcomes measured**
    Baseline 0m 1 to <3m 3 to <6m 6 to <12m 12 to<24m >24m
    Other (specify):
7. **Implementation fidelity/monitoring**:
8. Does a protocol exist for the trial? Yes No  unknown
9. Was the protocol obtained to assess the adherence of the trial? Yes No

From where can it be obtained?

1. Was participants’ involvement / compliance monitored Yes No  unknown
2. If yes, how was participants’ involvement / compliance monitored?

|  |
| --- |

1. **Incentives for participation**: Did the participants receive any incentives?

|  |
| --- |

1. **Quality assessment**
   - - 1. **COCHRANE QUALITY ASSESSMENT**
2. **Sequence generation** (was the allocation sequence adequately generated?):
    Yes No Unclear
3. **Allocation concealment** (was allocation adequately concealed?):
   Yes No Unclear
4. **Blinding** (was knowledge of the allocated intervention adequately prevented during the study?): 
   Yes No Unclear
5. **Incomplete outcome data** (were incomplete outcome data due to attrition and exclusions adequately addressed?):
   Yes No Unclear
6. **Selective reporting** (are reports of the study free of suggestion of selective outcome reporting?):
   Yes No Unclear
7. **Conflict of interest** declared:
   Yes No Unclear
   - - 1. **EPHPP QUALITY ASSESSMENT TOOL FOR QUANTITATIVE STUDIES**
8. **Selection Bias**
9. Are the individuals selected to participate in the study likely to be representative of the target population?
   Very likely somewhat likely not likely can’t tell
10. What percentage of selected individuals agreed to participate?
    80 - 100% 60 – 79% less than 60% not applicable can’t tell

RATINGS:

1. **Study Design**
2. Indicate the study design
   Cluster RCT RCT Controlled clinical trial Quasi RCT Factorial design Other
3. Was the study described as randomized? No Yes
4. If Yes, was the method of randomization described? No Yes
5. If Yes, was the method appropriate? No Yes

   RATINGS:
6. **Confounders**
7. Were there important differences between groups prior to the intervention?
   Yes No Can’t tell
   (Examples of confounders: Race; Sex; Marital status/family; Age; SES (income or class); Education; Health status; Pre-intervention score on outcome measure)
8. If yes, indicate the percentage of relevant confounders that were controlled (either in the design (e.g. stratification, matching) or analysis)?
   80 – 100% 60 – 79% Less than 60% Can’t Tell

   RATINGS:
9. **Blinding**
10. Was (were) the outcome assessor(s) aware of the intervention or exposure status of participants?
    Yes No Can’t tell
11. Were the study participants aware of the research question?
    Yes No Can’t tell

    RATINGS:
12. **Data Collection Methods**
13. Were data collection tools shown to be valid?
    Yes No Can’t tell
14. Were data collection tools shown to be reliable?
    Yes No Can’t tell

    RATINGS:
15. **Withdrawals And Drop-Outs**
16. Were withdrawals and drop-outs reported in terms of numbers and/or reasons per group?
    Yes No Can’t tell
17. Indicate the percentage of participants completing the study. (If the percentage differs by groups, record the lowest).
    80 -100% 60 - 79% less than 60% Can’t tell

    RATINGS:
18. **Intervention Integrity**
19. What percentage of participants received the allocated intervention or exposure of interest?
    80 -100% 60 - 79% less than 60% Can’t tell
20. Was the consistency of the intervention measured?
    Yes No Can’t tell
21. Is it likely that subjects received an unintended intervention (contamination or co-intervention) that may influence the results?
    Yes No Can’t tell
22. **Analyses**
23. Indicate the unit of allocation:
    community organization/institution practice/office individual
24. Indicate the unit of analysis:
    community organization/institution practice/office individual
25. Are the statistical methods appropriate for the study design?
    Yes No Can’t tell
26. Is the analysis performed by intervention allocation status (i.e. intention to treat) rather than the actual intervention received?
    Yes No Can’t tell

**GLOBAL RATING**

***COMPONENT RATINGS***

1. **Selection Bias**:
2. **Study Design**:
3. **Confounders**:
4. **Blinding**:
5. **Data Collection Methods**:
6. **Withdrawals And Dropouts**:
7. ***GLOBAL RATING FOR THIS PAPER***

**1-STRONG** (four STRONG ratings with no WEAK ratings)

**2-MODERATE** (less than four STRONG ratings and one WEAK rating)

**3-** **WEAK** (two or more WEAK ratings)

With both reviewers discussing the ratings:

Is there a discrepancy between the two reviewers with respect to the component (A-F) ratings?
No Yes

If yes, indicate the reason for the discrepancy
Oversight Differences in interpretation of criteria  Differences in interpretation of study

Final decision of both reviewers:
**1-STRONG** **2-MODERATE** **3-WEAK**

1. **PARTICIPANTS:**

**Eligibility criteria**:

|  |
| --- |

|  |  | **Intervention group I** | **Intervention group II** | **Comparator** |
| --- | --- | --- | --- | --- |
| Number of participants (Sample size) | n |  |  |  |
| Age | Mean (SD) |  |  |  |
| Gender | n (%) |  |  |  |
| Ethnicity | n (%) |  |  |  |
| Socio economic status | n (%) |  |  |  |
| Educational level | Cat (%) |  |  |  |
|  |  |  |  |  |
| Health status: |  |  |  |  |
| Obese | n (%) |  |  |  |
| Diabetic | n (%) |  |  |  |
| Hypertensive | n (%) |  |  |  |
| Smoker | n (%) |  |  |  |
| Other (Specify) |  |  |  |  |
|  |  |  |  |  |
| Internet access at home | n (%) |  |  |  |
| Weekly internet use, minutes | Mean (SD) |  |  |  |
| Technology experience, years | Mean (SD) |  |  |  |
|  |  |  |  |  |
| Psychological characteristics (specify) |  |  |  |  |

1. **INTERVENTION**:

|  |
| --- |

1. **Contents/components** (Describe the different components of the intervention)

Please specify if the intervention manual/CD/URL is available.

1. **Software** used for tailoring intervention techniques

|  |
| --- |

1. **Setting**

Home School/College Workplace Community centre Primary care Secondary care
 Not specified  Not applicable (ie internet site)  Other (specify):

1. **Mode of delivery**:
   Internet Mobile based CD-ROM Multimedia game  Other (specify):
2. **Media used** (e.g. picture, text, sound)

|  |
| --- |

1. **Duration of intervention**
2. **Duration :**
3. **Intensity:**
4. **Frequency:**
5. Describe **the comparator** (e.g. usual care) as described in the report

|  |
| --- |

1. **Intervention development**
2. Was the **intervention development informed by the theory**? Yes No Unclear
3. If yes, describe **how theory was used in the development of the intervention**?

|  |
| --- |

1. Was the **theory used to tailor intervention techniques** to recipients? Yes No Unclear
2. If yes, describe **how theory was used to tailor intervention techniques**?

|  |
| --- |

1. Mention the **theoretical constructs** (modifying factors, e.g. attitudes, self-efficacy, norms) that were described by the author

|  |
| --- |

1. **Measurement of the constructs** (specify how many were measured and how?)

|  |
| --- |

1. Were the **mediation effects of the constructs measured**? (If so how many and how?)

|  |
| --- |

1. **Theories of behaviour change** used

**Intervention Control**

Attribution theory

Cognitive adaptation theory

Cognitive behavioural theory

Decision making theory
Demand-control model
Diffusion theory

Effort-reward imbalance

Elaboration likelihood model

Fear arousal theory

Goal theory

Group theory

Health beliefs model

Implementation theory/automotive model
Intrinsic motivation theories

Locus of control theories

Operant learning theory

Person situation contingency models

Precaution adoption process model

Relapse prevention theory

Self-determination theory

Self-regulation theory

Social cognitive theory

Social comparison theory

Social-ecological theory

Social identity theory

Social influence

Social identity theory

Social learning theory

Theory of Planned Behaviour

Transtheoretical model

Volitional control theory

Other (specify):

Attribution theory

Cognitive adaptation theory

Cognitive behavioural theory

Decision making theory
Demand-control model
Diffusion theory

Effort-reward imbalance

Elaboration likelihood model

Fear arousal theory

Goal theory

Group theory

Health beliefs model

Implementation theory/automotive model
Intrinsic motivation theories

Locus of control theories

Operant learning theory

Person situation contingency models

Precaution adoption process model

Relapse prevention theory

Self-determination theory

Self-regulation theory

Social cognitive theory

Social comparison theory

Social-ecological theory

Social identity theory

Social influence

Social identity theory

Social learning theory

Theory of Planned Behaviour

Transtheoretical model

Volitional control theory

Other (specify):

1. Describe the **behaviour change techniques** used

**Intervention Control**

Action planning

Agree behavioural contract

Barrier identification/Problem solving

Emotional control training

Environmental restructuring

Facilitate social comparison

Fear Arousal

General communication skills training
Goal setting (behaviour)

Goal setting (outcome)

Model/ Demonstrate the behavior

Motivational interviewing

Plan social support/ social change

Prompt anticipated regret

Prompt identification as role model/ position advocate

Prompt practice

Prompt review of behavioural goals

Prompt review of outcome goals

Prompt Self talk

Prompt self-monitoring of behaviour

Prompt self-monitoring of behavioural outcome

Prompt use of imagery

Prompting focus on past success

Prompting generalisation of a target behavior

Provide feedback on performance

Provide information about others’ approval

Provide information on consequences of behaviour in general
Provide information on consequences of behaviour to the individual

Provide information on where and when to perform the behavior
Provide instruction on how to perform the behavior

Provide normative information about others’ behaviour

Provide rewards contingent on effort or progress towards behaviour

Provide rewards contingent on successful behaviour

Relapse prevention/ Coping planning

Set graded tasks

Shaping

Stress management

Teach to use prompts/ cues

Time management

Use of follow up prompts

Other (Specify)

Action planning

Agree behavioural contract

Barrier identification/Problem solving

Emotional control training

Environmental restructuring

Facilitate social comparison

Fear Arousal

General communication skills training
Goal setting (behaviour)

Goal setting (outcome)

Model/ Demonstrate the behavior

Motivational interviewing

Plan social support/ social change

Prompt anticipated regret

Prompt identification as role model/ position advocate

Prompt practice

Prompt review of behavioural goals

Prompt review of outcome goals

Prompt Self talk

Prompt self-monitoring of behaviour

Prompt self-monitoring of behavioural outcome

Prompt use of imagery

Prompting focus on past success

Prompting generalisation of a target behavior

Provide feedback on performance

Provide information about others’ approval

Provide information on consequences of behaviour in general
Provide information on consequences of behaviour to the individual

Provide information on where and when to perform the behavior
Provide instruction on how to perform the behavior

Provide normative information about others’ behaviour

Provide rewards contingent on effort or progress towards behaviour

Provide rewards contingent on successful behaviour

Relapse prevention/ Coping planning

Set graded tasks

Shaping

Stress management

Teach to use prompts/ cues

Time management

Use of follow up prompts

Other (Specify)

1. **OUTCOME MEASURES**
2. **Dietary Assessment (Primary outcomes)**
3. **Data Collection Methods**

Self-administered Interview-administered

1. **Data collection Modes**Postal Telephone Computer Face-to-face other (specify):
2. **Diet Assessment Tools/Techniques Used:**
3. **Estimated food record**; Validation Yes No
    If yes, for which nutrients:      
   Number of days:
4. **Weighed food record**; Validation Yes No
   If yes, for which nutrients:     
   Number of days:
5. **24-Hour Recall**; Validation Yes No
   If yes, for which nutrients:      
   Number of days:
6. **Diet History**; Validation Yes No
   If yes, for which nutrients:      
   Time period:
7. **Food Frequency Questionnaire** (FFQ):
   individual portion Small/medium/large portion average portion no portion

Validation: Yes No
If yes, for which nutrients:      
Time period:

1. **Clinical Assessment (Secondary outcomes)**
2. **Data Collection Methods**

Self-administered Interview-administered  Objectively measured

1. **Data collection Modes**Postal Telephone Computer Face-to-face other (specify):
2. **Clinical Assessment Tools/Techniques Used:**
3. **Anthropometric techniques**

Specify:

Validation Yes No

1. **Biochemical techniques**

Specify:

Validation Yes No

1. **Primary Outcomes**
2. **Energy & Nutrients**
   1. Baseline

| **Primary Outcomes** | **Units** | **Intervention Group I** | **Intervention Group II** | **Comparator** |
| --- | --- | --- | --- | --- |
| Energy | KJ/Kcal |  |  |  |
| Protein | g |  |  |  |
| Total Fat | g |  |  |  |
| Saturated Fat | g |  |  |  |
| Poly unsaturated Fat | g |  |  |  |
| Mono unsaturated Fat | g |  |  |  |
| % of energy from Fat | % |  |  |  |
| % energy Saturated Fat | % |  |  |  |
| Carbohydrate (CHO) | g |  |  |  |
| % of energy from CHO | % |  |  |  |
| Total Sugar | g |  |  |  |
| Sucrose | g |  |  |  |
| Dietary fibre | g |  |  |  |
| Non-starch polysaccharide fibre | g |  |  |  |
| Calcium | mg |  |  |  |
| Iron | mg |  |  |  |
| Zinc | mg |  |  |  |
| Sodium | mg |  |  |  |
| Vitamin A | RE/RAE/µg/ µmol/IU |  |  |  |
| Vitamin C | Mg |  |  |  |
| Vitamin E | mg |  |  |  |
| Vitamin B_9_ / Folate | µg |  |  |  |
| B_12_ | µg |  |  |  |
| Vitamin B_1_ / Thiamine | mg |  |  |  |
| Vitamin B_2_ / Riboflavin | mg |  |  |  |
| Vitamin B_3_ / Niacin | mg |  |  |  |
| Other |  |  |  |  |

- 1. Follow-up

| **Primary Outcomes** | **Units** | **Intervention Group I** | **Intervention Group II** | **Comparator** |
| --- | --- | --- | --- | --- |
| Energy | KJ/Kcal |  |  |  |
| Protein | g |  |  |  |
| Total Fat | g |  |  |  |
| Saturated Fat | g |  |  |  |
| Poly unsaturated Fat | g |  |  |  |
| Mono unsaturated Fat | g |  |  |  |
| % of energy from Fat | % |  |  |  |
| % energy Saturated Fat | % |  |  |  |
| Carbohydrate (CHO) | g |  |  |  |
| % of energy from CHO | % |  |  |  |
| Total Sugar | g |  |  |  |
| Sucrose | g |  |  |  |
| Dietary fibre | g |  |  |  |
| Non-starch polysaccharide fibre | g |  |  |  |
| Calcium | mg |  |  |  |
| Iron | mg |  |  |  |
| Zinc | mg |  |  |  |
| Sodium | mg |  |  |  |
| Vitamin A | RE/RAE/µg/ µmol/IU |  |  |  |
| Vitamin C | Mg |  |  |  |
| Vitamin E | mg |  |  |  |
| Vitamin B_9_ / Folate | µg |  |  |  |
| B_12_ | µg |  |  |  |
| Vitamin B_1_ / Thiamine | mg |  |  |  |
| Vitamin B_2_ / Riboflavin | mg |  |  |  |
| Vitamin B_3_ / Niacin | mg |  |  |  |
| Other |  |  |  |  |

1. **Prevalence Rates for Primary Outcome (%)**
   1. Baseline

| **Primary outcomes** | **Criteria used** | **Intervention Group I** | **Intervention Group II** | **Comparator** |
| --- | --- | --- | --- | --- |
| Energy |  |  |  |  |
| Protein |  |  |  |  |
| Total Fat |  |  |  |  |
| Saturated Fat |  |  |  |  |
| Poly unsaturated Fat |  |  |  |  |
| Mono unsaturated Fat |  |  |  |  |
| % of energy from Fat |  |  |  |  |
| % of energy Saturated Fat |  |  |  |  |
| Carbohydrate (CHO) |  |  |  |  |
| % of energy from CHO |  |  |  |  |
| Sugar |  |  |  |  |
| Sucrose |  |  |  |  |
| Dietary fibre |  |  |  |  |
| NSP fibre |  |  |  |  |
| Calcium |  |  |  |  |
| Iron |  |  |  |  |
| Zinc |  |  |  |  |
| Sodium |  |  |  |  |
| Vitamin A |  |  |  |  |
| Vitamin C |  |  |  |  |
| Vitamin E |  |  |  |  |
| Vitamin B_9_ / Folate |  |  |  |  |
| B_12_ |  |  |  |  |
| Vitamin B_1_ / Thiamine |  |  |  |  |
| Vitamin B_2_ / Riboflavin |  |  |  |  |
| Vitamin B_3_ / Niacin |  |  |  |  |
| Other |  |  |  |  |

- 1. **Follow-up**

| **Primary outcomes** | **Criteria used** | **Intervention Group I** | **Intervention Group II** | **Comparator** |
| --- | --- | --- | --- | --- |
| Energy |  |  |  |  |
| Protein |  |  |  |  |
| Total Fat |  |  |  |  |
| Saturated Fat |  |  |  |  |
| Poly unsaturated Fat |  |  |  |  |
| Mono unsaturated Fat |  |  |  |  |
| % of energy from Fat |  |  |  |  |
| % of energy Saturated Fat |  |  |  |  |
| Carbohydrate (CHO) |  |  |  |  |
| % of energy from CHO |  |  |  |  |
| Sugar |  |  |  |  |
| Sucrose |  |  |  |  |
| Dietary fibre |  |  |  |  |
| NSP fibre |  |  |  |  |
| Calcium |  |  |  |  |
| Iron |  |  |  |  |
| Zinc |  |  |  |  |
| Sodium |  |  |  |  |
| Vitamin A |  |  |  |  |
| Vitamin C |  |  |  |  |
| Vitamin E |  |  |  |  |
| Vitamin B_9_ / Folate |  |  |  |  |
| B_12_ |  |  |  |  |
| Vitamin B_1_ / Thiamine |  |  |  |  |
| Vitamin B_2_ / Riboflavin |  |  |  |  |
| Vitamin B_3_ / Niacin |  |  |  |  |
| Other |  |  |  |  |

1. **Food Group Outcomes:**
   1. Baseline

| **Food groups** | **Units** | **Intervention Group I** | **Intervention Group II** | **Comparator** |
| --- | --- | --- | --- | --- |
| Fruits (F) |  |  |  |  |
| Vegetables (V) |  |  |  |  |
| Juices (J) |  |  |  |  |
| FV |  |  |  |  |
| FJV |  |  |  |  |
| Dairy / Milk |  |  |  |  |
| Wholegrain Cereals |  |  |  |  |
| Refined cereals |  |  |  |  |
| Legumes |  |  |  |  |
| Nuts |  |  |  |  |
| Meat (M) |  |  |  |  |
| Poultry (P) |  |  |  |  |
| Fish (Fi) |  |  |  |  |
| Meat, Poultry, Fish |  |  |  |  |
| Eggs |  |  |  |  |
| Artificially Sweetened Beverages |  |  |  |  |
| Sugared beverages |  |  |  |  |
| Added Fat – Butter/Ghee |  |  |  |  |
| Added Fat – Oil |  |  |  |  |
| Added Fat – Margarine |  |  |  |  |
| Added sugars |  |  |  |  |
| Fast foods |  |  |  |  |
| Salt |  |  |  |  |
| Others |  |  |  |  |

- 1. **Follow-up**

| **Food groups** | **Units** | **Intervention Group I** | **Intervention Group II** | **Comparator** |
| --- | --- | --- | --- | --- |
| Fruits (F) |  |  |  |  |
| Vegetables (V) |  |  |  |  |
| Juices (J) |  |  |  |  |
| FV |  |  |  |  |
| FJV |  |  |  |  |
| Dairy / Milk |  |  |  |  |
| Wholegrain Cereals |  |  |  |  |
| Refined cereals |  |  |  |  |
| Legumes |  |  |  |  |
| Nuts |  |  |  |  |
| Meat (M) |  |  |  |  |
| Poultry (P) |  |  |  |  |
| Fish (Fi) |  |  |  |  |
| Meat, Poultry, Fish |  |  |  |  |
| Eggs |  |  |  |  |
| Artificially Sweetened Beverages |  |  |  |  |
| Sugared beverages |  |  |  |  |
| Added Fat – Butter/Ghee |  |  |  |  |
| Added Fat – Oil |  |  |  |  |
| Added Fat – Margarine |  |  |  |  |
| Added sugars |  |  |  |  |
| Fast foods |  |  |  |  |
| Salt |  |  |  |  |
| Others |  |  |  |  |

1. **Secondary Outcomes**
   - 1. **Anthropometric and biochemical**
        1. Baseline

| **Secondary Outcomes** | **Tools Used** | **Intervention Group I** | **Intervention Group II** | **Comparator** |
| --- | --- | --- | --- | --- |
| **Anthropometric** | | | | |
| BMI |  |  |  |  |
| % Body Fat |  |  |  |  |
| Other |  |  |  |  |
| **Biochemical - Lipids** | | | | |
| Total Cholesterol |  |  |  |  |
| HDL |  |  |  |  |
| LDL |  |  |  |  |
| Ratio HDL/LDL |  |  |  |  |
| Triglycerides |  |  |  |  |
| **Biochemical - Other** | | | | |
| Ascorbic Acid |  |  |  |  |
| Serum Folate |  |  |  |  |
| Red Blood Cell Folate |  |  |  |  |
| Homocysteine |  |  |  |  |
| Haemoglobin |  |  |  |  |
| Ferritin |  |  |  |  |
| Urinary iodine |  |  |  |  |
| Urinary Sodium |  |  |  |  |
| Retinol |  |  |  |  |
| Carotenoids |  |  |  |  |
| Α-Tocopherol |  |  |  |  |
| Plasma/Serum Zinc |  |  |  |  |
| Other |  |  |  |  |

- - - 1. **Follow-up**

| **Secondary Outcomes** | **Tools Used** | **Intervention Group I** | **Intervention Group II** | **Comparator** |
| --- | --- | --- | --- | --- |
| **Anthropometric** | | | | |
| BMI |  |  |  |  |
| % Body Fat |  |  |  |  |
| Other |  |  |  |  |
| **Biochemical - Lipids** | | | | |
| Total Cholesterol |  |  |  |  |
| HDL |  |  |  |  |
| LDL |  |  |  |  |
| Ratio HDL/LDL |  |  |  |  |
| Triglycerides |  |  |  |  |
| **Biochemical - Other** | | | | |
| Ascorbic Acid |  |  |  |  |
| Folate |  |  |  |  |
| Red Blood Cell Folate |  |  |  |  |
| Homocysteine |  |  |  |  |
| Haemoglobin |  |  |  |  |
| Serum Ferritin |  |  |  |  |
| Urinary iodine |  |  |  |  |
| Urinary Sodium |  |  |  |  |
| Retinol |  |  |  |  |
| Carotenoids |  |  |  |  |
| Α-Tocopherol |  |  |  |  |
| Plasma/Serum Zinc |  |  |  |  |
| Other |  |  |  |  |

- - 1. **Prevalence Rates for Secondary Outcomes**
       1. Baseline

| **Secondary Outcomes** | **Criteria Used** | **Intervention Group I** | **Intervention Group II** | **Comparator** |
| --- | --- | --- | --- | --- |
| **Anthropometric** | | | | |
| Underweight |  |  |  |  |
| Normal weight |  |  |  |  |
| Overweight |  |  |  |  |
| Obese |  |  |  |  |
| % Body Fat |  |  |  |  |
| Others |  |  |  |  |
| **Biochemical -Lipids** | | | | |
| Total Cholesterol |  |  |  |  |
| HDL |  |  |  |  |
| LDL |  |  |  |  |
| Ratio HDL/LDL |  |  |  |  |
| Triglycerides |  |  |  |  |
| **Biochemical - other** | | | | |
| Ascorbic Acid |  |  |  |  |
| Serum Folate |  |  |  |  |
| Red Blood Cell Folate |  |  |  |  |
| Homocysteine |  |  |  |  |
| Haemoglobin |  |  |  |  |
| Ferritin – iron deficiency anaemia |  |  |  |  |
| Ferritin – iron deficiency without anaemia |  |  |  |  |
| Urinary iodine |  |  |  |  |
| Urinary Sodium |  |  |  |  |
| Retinol |  |  |  |  |
| Carotenoids |  |  |  |  |
| Α-Tocopherol |  |  |  |  |
| Plasma/Serum Zinc |  |  |  |  |
| Other |  |  |  |  |

- - - 1. **Follow-up**

| **Secondary Outcomes** | **Criteria Used** | **Intervention Group I** | **Intervention Group II** | **Comparator** |
| --- | --- | --- | --- | --- |
| **Anthropometric** | | | | |
| Underweight |  |  |  |  |
| Normal weight |  |  |  |  |
| Overweight |  |  |  |  |
| Obese |  |  |  |  |
| % Body Fat |  |  |  |  |
| Others |  |  |  |  |
| **Biochemical -Lipids** | | | | |
| Total Cholesterol |  |  |  |  |
| HDL |  |  |  |  |
| LDL |  |  |  |  |
| Ratio HDL/LDL |  |  |  |  |
| Triglycerides |  |  |  |  |
| **Biochemical - other** | | | | |
| Ascorbic Acid |  |  |  |  |
| Folate |  |  |  |  |
| Red Blood Cell Folate |  |  |  |  |
| Homocysteine |  |  |  |  |
| Haemoglobin |  |  |  |  |
| Ferritin – iron deficiency anaemia |  |  |  |  |
| Ferritin – iron deficiency without anaemia |  |  |  |  |
| Urinary iodine |  |  |  |  |
| Urinary Sodium |  |  |  |  |
| Retinol |  |  |  |  |
| Carotenoids |  |  |  |  |
| Α-Tocopherol |  |  |  |  |
| Plasma/Serum Zinc |  |  |  |  |
| Other |  |  |  |  |

1. **Mechanisms of change** (only when primary or secondary outcomes are reported)

| **Mediators** | **Tools used** | **Intervention Group I** | **Intervention Group II** | **Comparator** |
| --- | --- | --- | --- | --- |
| 1. **Cognitive mediators** | | | | |
| Knowledge |  |  |  |  |
| Self-efficacy |  |  |  |  |
| Intention |  |  |  |  |
| Other |  |  |  |  |
| 1. **Emotional mediators** | | | | |
| Anxiety/depression |  |  |  |  |
| Stress |  |  |  |  |
| Wellbeing/happiness |  |  |  |  |
| Other |  |  |  |  |
| 1. **Antecedents** | | | | |
| Specify |  |  |  |  |
| 1. **Consequences** | | | | |
| Specify |  |  |  |  |
| 1. **Organisational / environmental influences** | | | | |
| Specify |  |  |  |  |

1. **Additional Measures of Engagement**

| **Additional measures** | **Units** | **Intervention Group I** | **Intervention Group II** | **Comparator** |
| --- | --- | --- | --- | --- |
| Log on rates |  |  |  |  |
| Number of messages posted to the discussion group |  |  |  |  |
| Pages visited |  |  |  |  |
| Other measures of compliance (specify) |  |  |  |  |

1. **Further Comments (Eg- process):**

|  |
| --- |

1. Specify **whether the above findings were explained in relation to theory, or theories, on which the interventions was based**?

|  |
| --- |
