# Supplementary material for: Assessing the effectiveness and cost effectiveness of adaptive e-Learning to improve dietary behaviour: protocol for a systematic review
Source: BMC Public Health. 2010 Apr 21;10:200. doi: 10.1186/1471-2458-10-200 (PMC2868000; doi:10.1186/1471-2458-10-200)
Supplement: Additional file 5 — Data extraction manual (Word file). A manual to guide data extraction. [file 1471-2458-10-200-S5.DOC]

**Review author**: Enter the name of the person extracting the data.
**Date**: Enter the date of extraction.

**Author:** Name the trials first author’s last name. (For example, if the trial is Block, G., Miller, M., Harnack, L., Kayman, S., Mandel, S. and Cristofar, S. (2000). An interactive CD-ROM for nutrition screening and counselling. American Journal of Public Health, 90, 781-785, then enter **Block**).

**Year of publication:** Enter the year of publication of the trial in the peer-review journal. In the above example, it would be **2000.** If the report is a grey literature (unpublished work) then enter the year that appears on the report.

**Country**: Specify the country where the intervention takes place.

**Title:** Name the title as it appears in the journal**.**  E.g. ‘**An interactive CD-ROM for nutrition screening and counselling’.**

**Aims & Objectives:** Report the general questions the trial was designed to answer. It may be associated with one or more hypotheses that, when tested, would help answer the question.

1. **METHODS**
2. **Recruitment** (It is the process of getting participants into a randomized trial)(1-2)***:***
3. **Electronic:** It is also called as internet or online recruitment where information technology (IT) is used for identifying and enrolling the participants.
4. **Mail shot**: Participants are invited by postal mail along with the interest survey, and if interested, would return the interest survey by post. e.g.- Using random mailings
5. **Telephone:** The interviewer calls the participants and conducts the screening by phone. e.g.- cold calls, Computer assisted telephone interviews/ Interactive voice response techniques.
6. **Advert in clinic:** e.g.- fliers, brochures etc.
7. **Advert elsewhere:** Media advertising using television and/ or newspapers, press releases/public service announcements, posting fliers
8. **Social media networks**: e.g.- Facebook, Twitter etc.
9. **Word of mouth**, if the information about enrolling for the trial passed from person to person.
10. **Physician or health worker referral:** Physician / GP or other registered health worker refers the participant to the trial.
11. **Organisation referral**: If an organisation such as a health insurance company refers the participant to the trial
12. **Other:** If other, please specify
13. **Study Design**(3)**:**
14. **Parallel-group trial**s: Patients are randomised to the new treatment or to the standard treatment and followed-up to determine the effect of each treatment in parallel groups.
15. **Crossover trials**: Patients are randomised to different sequences of treatments, but all patients eventually all treatments in varying order, i.e., the patient is his/her own control.
16. **Cluster randomised trials**: the unit of randomisation is at the group level where randomisation of individuals is inappropriate. Examples of clusters are villages, factories or schools.
17. **Factorial trials**: They assign patients to more than one treatment-comparison group. These are randomised in one trial at the same time, i.e., while drug A is being tested against placebo, patients are re-randomised to drug B or placebo, making four possible treatment combinations in total.
18. **Randomisation - Sequence generation** (The procedure used to obtain the (random) sequence for making intervention assignments)(4-5):
19. **Simple randomisation, tossing a coin**: heads may mean allocation to treatment A and tails to treatment B. Using randomisation lists prepared by this method ensures each patient has an equal chance of either treatment with no connection between assignments.
20. **Simple randomisation, random number table**: A random number table is a listing of random numbers (for example, in a sequence of 100 randomised numbers from 0 -9, the numbers 0-4 is allocated to treatment A and 5-9 to treatment B.
21. **Simple randomisation, computerised random number generator:** A random number generator is a process that produces [random numbers](http://stattrek.com/Tables/Random.aspx#randomnum) using a statistical algorithm (for example, The Random Number Generator produces a Random Number Table consisting of 10 entries, where each entry is the number 1 or 2. The researchers then assign one intervention to volunteer number 1, the second to volunteer number 2, and so on.
22. **Random permuted blocks**: here the number of patients allocated to each treatment is the same at certain points in the recruitment process. For example if a block size of 10 is used, then for each 'block' of 10 patients, five will be allocated to each treatment with the sequence of treatment allocations within each block ordered randomly.
23. **Stratified randomisation** is when randomisation with restriction is carried out separately within each of two or more subsets of participants (for example, defining disease severity or study centres) to ensure that the patient characteristics are closely balanced within each intervention group.
24. **Minimisation** is a method to ensure close balance between treatment groups for several patient factors. The principle behind minimisation is that the treatment allocation for a particular patient is determined by the characteristics of the patients already entered into the trial (with the first patient allocated treatment using simple randomisation).
25. **Response-adaptive designs**: There is a probability of being randomised to treatment in favour of the treatment that is showing more beneficial results so far for some outcome. For example, suppose that there are two treatment arms and that at the start of the study the probability of being randomised to either treatment group is equal (i.e. 50:50). As data accrue, one treatment is likely to show more favourable results, however small this benefit may seem. In a response-adaptive design, new participants would therefore be randomised to the treatment doing better with probability more than ½.
26. **Randomised consent design (Zelen design)**: when patients are randomised to one of two groups depending on consent- a) a group where no consent is asked and is assigned the standard group A and b) a group where patients are asked whether they are willing to accept the new treatment B. Patients unwilling to accept B are assigned to A.
27. **Randomization - Allocation concealment** ([Method used to implement the random allocation sequence](http://www.consort-statement.org/index.aspx?o=1026) (*e.g*., numbered containers or central telephone), clarifying whether the sequence was concealed until interventions were assigned (6-7).
28. **Centralised randomisation by telephone - automated:** With this method, an investigator rings a central telephone number once the patient has given informed consent, gives the patient's details which register the patient before the allocation is given, and only then is the patient's allocated treatment group revealed. This '[allocation concealment](javascript:glossary('allocation_concealment'))' greatly reduces the risk of selection bias arising from the randomisation being subverted. A telephone randomisation service can be used both for randomisation with a fixed list (such as simple or stratified randomisation), or minimisation (where, treatments are assigned as patients enter the trial). The treatment assignment is done electronically (automated).
29. **Centralised randomisation by telephone – person:** Same as above except that the treatment assignment is done by a person.
30. **Web-based randomisation:** Investigators randomize patients with only a few clicks by simply completing an on-screen form with patient details and are immediately notified of the treatment allocation.
31. **Sequentially numbered, sealed envelopes:** Here, as patients give informed consent and are entered into the trial, the investigator opens the next in a set of sequentially numbered sealed envelopes. Inside the envelope is the treatment assignment. The investigator may then be required to fax, send or email the patient's details to the trial coordinating centre if there is one.
32. **Sealed envelopes not sequentially numbered or not opaque**
33. **Random numbers read by someone not entering patients into trial (closed list)**
34. **Random numbers read by someone entering patients into trial (open list)**
35. **Randomization –Implementation** (6)
36. Who **generated** the allocation concealment?
37. Who **enrolled** participants?
38. [Who **assigned** participants to their groups](http://www.consort-statement.org/index.aspx?o=1027)?
39. **Analysis**
40. **Intention to treat analysis**: A strategy for analyzing data in which all participants are included in the group to which they were assigned, whether or not they completed the intervention given to the group.
    Intention-to-treat analysis prevents bias caused by the loss of participants, which may disrupt the baseline equivalence established by random assignment and which may reflect non-adherence to the protocol.
41. **Per protocol analysis:** An analysis restricted to patients who adhered to their assigned treatment in a randomized trial (omitting patients who dropped out of the study or for other reasons did not actually receive the planned intervention).
42. **Times at which outcomes measured:**

    **Baseline**: This consists of the data that are collected before randomisation. E.g., age, sex, symptoms etc.
    **0m**: The data are collected immediately following the intervention.
    **1m /1 to <3m/3 to <6m/6 to <12m/12 to<24m/>24m**: corresponds to the months when data were collected.
    **Other**: Mention if data was collected at times other than the above. If other, please specify
43. **Implementation fidelity/monitoring** (8)**:**
44. Does a **protocol exist** for the trial?

*Indicate whether a protocol is known / confirmed to exist for this trial*

1. Was the **protocol obtained to assess the adherence** of the trial?
   *If a protocol exists for the trial, indicate if and where it can be accessed (e.g., Web address), and, if available, provide registration information including registration number.*
2. Was participants’ **involvement/compliance** monitored?

*‘compliance refers to a parameter indicative of the quality and/or performance of a participant in a clinical trial, generally indicated by adherence to protocol, such as adherence to set procedures in a clinical trial, adherence to or achievement of certain goals of a clinical trial, such as enrolment goals, and/or any other parameter indicative of overall performance of a participant in a clinical trial’*

1. If yes, how was participants’ **involvement / compliance** monitored?

*E.g. reviewing participant’s log-on data or assigning a personal password to each participant to monitor their website login frequency and use.*

1. **Incentives for participation**: Did the participants receive any incentives (e.g. lottery system to win $20 gift cards in Student bodies 2 – Doyle 2008)?
2. **Quality assessment

   i. COCHRANE QUALITY ASSESSMENT**

The Cochrane Collaboration’s tool for assessing risk of bias (1-5) (9)

1. **Sequence generation** (was the allocation sequence adequately generated?): Describe the method used to generate the allocation sequence in sufficient detail to allow an assessment of whether it should produce comparable groups.
   Yes No Unclear
2. **Allocation concealment** (was allocation adequately concealed?): Describe the method used to conceal the allocation sequence in sufficient detail to determine whether intervention allocations could have been foreseen in advance of, or during, enrolment.
   Yes No Unclear
3. **Blinding** (was knowledge of the allocated intervention adequately prevented during the study?): Describe all measures used, if any, to blind study participants and personnel from knowledge of which intervention a participant received. Provide any information relating to whether the intended blinding was effective.
   Yes No Unclear
4. **Incomplete outcome data** (were incomplete outcome data due to attrition and exclusions adequately addressed?): Describe the completeness of outcome data for each main outcome, including attrition and exclusions from the analysis. State whether attrition and exclusions were reported, the numbers in each intervention group (compared with total randomized participants), reasons for attrition/exclusions where reported, and any re-inclusions in analyses performed by the review authors.
   Yes No Unclear
5. **Selective reporting** (are reports of the study free of suggestion of selective outcome reporting?): State how the possibility of selective outcome reporting was examined by the review authors, and what was found.
   Yes No Unclear
6. **Conflict of interest declared**:
   Yes No Unclear
7. **QUALITY ASSESSMENT TOOL FOR QUANTITATIVE STUDIES – EPHPP**

(10-11)

**COMPONENT RATINGS**

1. **Selection Bias**
2. Are the individuals selected to participate in the study likely to be representative of the target population?

*Participants are more likely to be representative of the target population if they are randomly selected from a comprehensive list of individuals in the target population (****score very likely****). They may not be representative if they are referred from a source (e.g. clinic) in a systematic manner (****score somewhat likely****) or self-referred (****score not likely****).*
 Very likely  somewhat likely  not likely  can’t tell

1. What percentage of selected individuals agreed to participate?

*Refers to the* ***%*** *of subjects in the control and intervention groups that agreed to participate in the study before they were assigned to intervention or control groups.* 80 - 100% agreement 60 – 79% agreement less than 60% agreement not applicable
 can’t tell

 RATINGS:

***Strong****: The selected individuals are very likely to be representative of the target population* ***(Q1 is 1)*** *and there is greater than 80% participation* ***(Q2 is 1)****.*

***Moderate****: The selected individuals are at least somewhat likely to be representative of the target population* ***(Q1 is 1 or 2)****; and there is 60 - 79% participation* ***(Q2 is 2)****. ‘Moderate’ may also be assigned if* ***Q1 is 1 or 2 and Q2 is 5*** *(can’t tell).*

***Weak****:**The selected individuals are not likely to be representative of the target population* ***(Q1 is 3)****;* ***or*** *there is less than 60% participation* ***(Q2 is 3)******or*** *selection is not described* ***(Q1 is 4)****; and the level of participation is not described* ***(Q2 is 5)****.*

1. **Study Design**
2. Indicate the study design
   Cluster RCT RCT Controlled clinical trial Quasi RCT Factorial design Other
3. Was the study described as randomized?

***Score YES****, if the authors used words such as random allocation, randomly assigned, and random assignment.*

***Score NO****, if no mention of randomization is made.*
 No Yes

1. If Yes, was the method of randomization described?

***Score YES****, if the authors describe any method used to generate a random allocation sequence.*

***Score NO****, if the authors do not describe the allocation method or describe methods of allocation such as alternation, case record numbers, dates of birth, day of the week, and any allocation procedure that is entirely transparent before assignment, such as an open list of random numbers of assignments. If NO is scored, then the study is a controlled clinical trial.* No Yes

1. If Yes, was the method appropriate?

***Score YES****, if the randomization sequence allowed each study participant to have the same chance of receiving each intervention and the investigators could not predict which intervention was next. Examples of appropriate approaches include assignment of subjects by a central office unaware of subject characteristics, or sequentially numbered, sealed, opaque envelopes.*

***Score NO****, if the randomization sequence is open to the individuals responsible for recruiting and allocating participants or providing the intervention, since those individuals can influence the allocation process, either knowingly or unknowingly.*

*If* ***NO is scored****, then the study is a controlled clinical trial.*

***Controlled Clinical Trial (CCT)*** *An experimental study design where the method of allocating study subjects to intervention or control groups is open to individuals responsible for recruiting subjects or providing the intervention. The method of allocation is transparent before assignment, e.g. an open list of random numbers or allocation by date of birth, etc.* No Yes

 RATINGS:

***Strong****: will be assigned to those articles that described RCTs and CCTs.*

***Moderate****: will be assigned to those that described a cohort analytic study, a case control study, a cohort design, or an interrupted time series.*

***Weak****: will be assigned to those that used any other method or did not state the method used.*

1. **Confounders**
2. Were there important differences between groups prior to the intervention?

*By definition, a* ***confounder*** *is a variable that is associated with the intervention or exposure and causally related to the outcome of interest. Even in a robust study design, groups may not be balanced with respect to important variables prior to the intervention. The authors should indicate if confounders were controlled in the design (by stratification or matching) or in the analysis. If the allocation to intervention and control groups is randomized, the authors must report that the groups were balanced at baseline with respect to confounders (either in the text or in a table).* Yes No Can’t tell
(Examples of confounders: Race; Sex; Marital status/family; Age; SES (income or class); Education; Health status; Pre-intervention score on outcome measure)

1. If yes, indicate the percentage of relevant confounders that were controlled (either in the design (e.g. stratification, matching) or analysis)?
   80 – 100% 60 – 79% Less than 60% Can’t Tell

RATINGS:

***Strong****: will be assigned to those articles that controlled for at least 80% of relevant confounders (Q1 is 2); or (Q2 is 1).*

***Moderate****: will be given to those studies that controlled for 60 – 79% of relevant confounders (Q1 is 1) and (Q2 is 2).*

***Weak****: will be assigned when less than 60% of relevant confounders were controlled (Q1 is 1) and (Q2 is 3) or control of confounders was not described (Q1 is 3) and (Q2 is 4).*

1. **Blinding**
2. Was (were) the outcome assessor(s) aware of the intervention or exposure status of participants?

*Assessors should be described as blinded to which participants were in the control and intervention groups. The purpose of blinding the outcome assessors (who might also be the care providers) is to protect against detection bias*.
 Yes No Can’t tell

1. Were the study participants aware of the research question?

*Study participants should not be aware of (i.e. blinded to) the research question. The purpose of blinding the participants is to protect against reporting bias.* Yes No Can’t tell

 RATINGS:

***Strong:*** *The outcome assessor is not aware of the intervention status of participants (Q1 is 2); and the study participants are not aware of the research question (Q2 is 2).*

***Moderate:*** *The outcome assessor is not aware of the intervention status of participants (Q1 is 2); or the study participants are not aware of the research question (Q2 is 2); or blinding is not described (Q1 is 3 and Q2 is 3).*

***Weak:*** *The outcome assessor is aware of the intervention status of participants (Q1 is 1); and the study participants are aware of the research question (Q2 is 1).*

1. **Data Collection Methods**

*Tools for primary outcome measures must be described as reliable and valid. If ‘face’ validity or ‘content’ validity has been demonstrated, this is acceptable. Some sources from which data may be collected are described below:*

*Self reported data includes data that is collected from participants in the study (e.g. completing a questionnaire, survey, answering questions during an interview, etc.).*

*Assessment/Screening includes objective data that is retrieved by the researchers. (e.g. observations by investigators).*

*Medical Records/Vital Statistics refers to the types of formal records used for the extraction of the data.*

*Reliability and validity can be reported in the study or in a separate study. For example, some standard assessment tools have known reliability and validity.*

1. Were data collection tools shown to be valid?
   Yes No Can’t tell
2. Were data collection tools shown to be reliable?
   Yes No Can’t tell

   RATINGS:

***Strong:*** *The data collection tools have been shown to be valid (Q1 is 1); and the data collection tools have been shown to be reliable (Q2 is 1).*

***Moderate:*** *The data collection tools have been shown to be valid (Q1 is 1); and the data collection tools have not been shown to be reliable (Q2 is 2) or reliability is not described (Q2 is 3).*

***Weak:*** *The data collection tools have not been shown to be valid (Q1 is 2) or both reliability and validity are not described (Q1 is 3 and Q2 is 3).*

1. **Withdrawals And Drop-Outs**
2. Were withdrawals and drop-outs reported in terms of numbers and/or reasons per group?

*Score* ***YES*** *if the authors describe BOTH the numbers and reasons for withdrawals and drop-outs.Score* ***NO*** *if either the numbers or reasons for withdrawals and drop-outs are not reported.* Yes No Can’t tell

1. Indicate the percentage of participants completing the study. (If the percentage differs by groups, record the lowest).

*The percentage of participants completing the study refers to the % of subjects remaining in the study at the final data collection period in all groups (i.e. control and intervention groups).*
 80 -100% 60 - 79% less than 60% Can’t tell

 RATINGS:

***Strong:*** *will be assigned when the follow-up rate is 80% or greater (Q2 is 1).*

***Moderate:*** *will be assigned when the follow-up rate is 60 – 79% (Q2 is 2) OR Q2 is 5 (N/A).*

***Weak:*** *will be assigned when a follow-up rate is less than 60% (Q2 is 3) or if the withdrawals and drop-outs were not*

*described (Q2 is 4).*

1. **Intervention Integrity**

*The number of participants receiving the intended intervention should be noted (consider both frequency and intensity). For example, the authors may have reported that at least 80 percent of the participants received the complete intervention. The authors should describe a method of measuring if the intervention was provided to all participants the same way. As well, the authors should indicate if subjects received an unintended intervention that may have influenced the outcomes. For example, co-intervention occurs when the study group receives an additional intervention (other than that intended). In this case, it is possible that the effect of the intervention may be over-estimated. Contamination refers to situations where the control group accidentally receives the study intervention. This could result in an under-estimation of the impact of the intervention.*

1. What percentage of participants received the allocated intervention or exposure of interest?
   80 -100% 60 - 79% less than 60% Can’t tell
2. Was the consistency of the intervention measured?
   Yes No Can’t tell
3. Is it likely that subjects received an unintended intervention (contamination or co-intervention) that may influence the results?
   Yes No Can’t tell
4. **Analyses**

*Was the quantitative analysis appropriate to the research question being asked?*

*An intention-to-treat analysis is one in which all the participants in a trial are analyzed according to the intervention to which they were allocated, whether they received it or not. Intention-to-treat analyses are favoured in assessments of effectiveness as they mirror the noncompliance and treatment changes that are likely to occur when the intervention is used in practice, and because of the risk of attrition bias when participants are excluded from the analysis.*

1. Indicate the unit of allocation:
   community organization/institution practice/office individual
2. Indicate the unit of analysis:
   community organization/institution practice/office individual
3. Are the statistical methods appropriate for the study design?
   Yes No Can’t tell
4. Is the analysis performed by intervention allocation status (i.e. intention to treat) rather than the actual intervention received?
   Yes No Can’t tell

**GLOBAL RATING**

***COMPONENT RATINGS***

1. **Selection Bias**:

*Strong: The selected individuals are very likely to be representative of the target population (Q1 is 1) and there is greater than 80% participation (Q2 is 1).*

*Moderate: The selected individuals are at least somewhat likely to be representative of the target population (Q1 is 1 or 2); and there is 60 - 79% participation (Q2 is 2). ‘Moderate’ may also be assigned if Q1 is 1 or 2 and Q2 is 5 (can’t tell).*

*Weak: The selected individuals are not likely to be representative of the target population (Q1 is 3); or there is less than 60% participation (Q2 is 3) or selection is not described (Q1 is 4); and the level of participation is not described (Q2 is 5).*

1. **Study Design**:

*Strong: will be assigned to those articles that described RCTs and CCTs.*

*Moderate: will be assigned to those that described a cohort analytic study, a case control study, a cohort design, or an interrupted time series.*

*Weak: will be assigned to those that used any other method or did not state the method used.*

1. **Confounders**:

*Strong: will be assigned to those articles that controlled for at least 80% of relevant confounders (Q1 is 2); or (Q2 is 1).*

*Moderate: will be given to those studies that controlled for 60 – 79% of relevant confounders (Q1 is 1) and (Q2 is 2).*

*Weak: will be assigned when less than 60% of relevant confounders were controlled (Q1 is 1) and (Q2 is 3) or control of confounders was not described (Q1 is 3) and (Q2 is 4).*

1. **Blinding**:

*Strong: The outcome assessor is not aware of the intervention status of participants (Q1 is 2); and the study participants are not aware of the research question (Q2 is 2).*

*Moderate: The outcome assessor is not aware of the intervention status of participants (Q1 is 2); or the study participants are not aware of the research question (Q2 is 2); or blinding is not described (Q1 is 3 and Q2 is 3).*

*Weak: The outcome assessor is aware of the intervention status of participants (Q1 is 1); and the study participants are aware of the research question (Q2 is 1).*

1. **Data Collection Methods**:

*Strong: The data collection tools have been shown to be valid (Q1 is 1); and the data collection tools have been shown to be reliable (Q2 is 1).*

*Moderate: The data collection tools have been shown to be valid (Q1 is 1); and the data collection tools have not been shown to be reliable (Q2 is 2) or reliability is not described (Q2 is 3).*

*Weak: The data collection tools have not been shown to be valid (Q1 is 2) or both reliability and validity are not described (Q1 is 3 and Q2 is 3).*

1. **Withdrawals And Dropouts**:

*Strong: will be assigned when the follow-up rate is 80% or greater (Q2 is 1).*

*Moderate: will be assigned when the follow-up rate is 60 – 79% (Q2 is 2) OR Q2 is 5 (N/A).*

*Weak: will be assigned when a follow-up rate is less than 60% (Q2 is 3) or if the withdrawals and drop-outs were not described (Q2 is 4).*

**Global Rating for This Paper**

**1-STRONG** (four STRONG ratings with no WEAK ratings)

**2-MODERATE** (less than four STRONG ratings and one WEAK rating)

**3 WEAK** (two or more WEAK ratings)

With both reviewers discussing the ratings:

Is there a discrepancy between the two reviewers with respect to the component (A-F) ratings?
No Yes

If yes, indicate the reason for the discrepancy
Oversight Differences in interpretation of criteria  Differences in interpretation of study

Final decision of both reviewers:
**1-STRONG** **2-MODERATE** **3-WEAK**

1. **PARTICIPANTS:** (Extract all the baseline data reported in the article. If needed, add additional columns or rows to incorporate the extra data).

**Eligibility criteria:** Specify the clinical and demographic characteristics that define those participants eligible to be enrolled in the trial.

|  |  | **Intervention group I** | **Intervention group II** | **Comparator** |
| --- | --- | --- | --- | --- |
| Number of participants (Sample size) | n | Report the number of participants allocated to each group following randomisation. | | |
| Age | Mean (SD) | Report the mean age in years for each of the group, and standard deviation if given. | | |
| Gender | n (%) | Report the % of male and female participants in each group. | | |
| Ethnicity | n (%) | Report the % of participants in any ethnic groups given. e.g. White, Mixed Race, Pakistani, Indian etc. | | |
| Socio economic status | n (%) | A combined measure of an individual's or household’s economic and social position relative to others, based on [income](http://en.wikipedia.org/wiki/Income), [education](http://en.wikipedia.org/wiki/Education), and [occupation](http://en.wikipedia.org/wiki/List_of_occupations). Report the % of participants in each of the SES groups given. | | |
| Educational level | Cat (%) | Report the % of participants at each education level. | | |
| Health status: | n (%) | Report the % of participants in each group with specific clinical conditions or risk factors. | | |
| Internet access at home | n (%) | Report the % of participants having Internet access at home. Participants using mobile Internet would also be included. | | |
| Weekly internet use, minutes | Mean (SD) | Report the average weekly Internet usage in minutes. | | |
| Technology experience, years | Mean (SD) | Report the average years of experience in technology, such as computer, mobile phone etc. | | |
| Psychological characteristics (specify) |  | Report the psychological characteristics of the groups, if given. E.g. stage of change; help seekers vs non-help seekers. | | |

1. **INTERVENTION** (The treatment or other health care course of action under investigation):
2. **Contents/components** (Describe the different components of the intervention)(8): (e.g. Programme component of Student Bodies 2 – Psycho educational readings; Discussion groups; Food, activity, and weight monitoring; Body image journal; Weekly newsletter (e-mailed); Personal profiles )

Please specify if the intervention manual/URL is available.

1. **Software used for tailoring intervention techniques**: Name the software as described in the paper. E.g. Squire’s Quest!
2. **Setting:**
   - 1. Home: If the intervention takes place where the participants live/reside.
     2. **School/College**: If the intervention takes place at an educational institution.
     3. **Workplace**: If the intervention takes place at the participant’s place of employment.
     4. **Community centre**: If the intervention takes place at public locations where members of a [community](http://en.wikipedia.org/wiki/Community) may gather for group activities, social support, public information, and other purposes (for example, Youth clubs).
     5. **Primary care**: If the intervention takes place at healthcare that is offered in the community and not in hospitals or specialist centres. General practitioners/family doctors are examples of healthcare professionals who work in primary care.
     6. **Secondary Care**: If the interventions takes place at hospitals or specialist centres where the services are provided by medical specialists who generally do not have first contact with [patients](http://en.wikipedia.org/wiki/Patients), for example, [cardiologists](http://en.wikipedia.org/wiki/Cardiologists), [urologists](http://en.wikipedia.org/wiki/Urologists) and dermatologists.
     7. **Not specified**: If the intervention setting was not specified
     8. **Not applicable**: If the intervention was not designed for a particular setting (for instance an online intervention that could be accessed from computers in the workplace, at home or elsewhere)
     9. **Other:** If other, please specify.
3. **Mode of delivery** (Describe how the intervention was delivered)(8):
4. **Internet**: If the intervention was web-based, that requires participants to have a computer with a broadband or a [dial-up connection](http://en.wikipedia.org/wiki/Dial-up_Internet_access).
5. **Mobile** based: If the intervention was web-based and delivered using Smartphone’s such as iphone, Blackberry.
6. **CD-ROM**: Interventions that use video disks.
7. **Multimedia game**: Interventions that use incorporate multiple forms of information content and processing such as digital integration of text, graphics, animation, audio, still images and motion video in a way that provides individual users with high levels of control and interaction (e.g.- Squire’s Quest! a ten-session, psychoeducational, multimedia game).
8. **Other**If other, please specify:
9. **Media used**: Mention the tools used to deliver the information or data (e.g. - audio narration, graphics).
10. **Duration of intervention**(8)
11. **Duration**: How long did the intervention last? (e.g. one session only; or sessions over 6 months).
12. **Intensity**: How long was each session? (e.g. - each session in Squire’s Quest! takes 25 minutes to complete).
13. **Frequency**: How many sessions were scheduled over the intervention? (e.g.- Squire’s Quest! has 10 sessions).
14. **Describe the comparator** **against which the intervention was tested** (e.g. - Usual care, or no intervention).
15. **Intervention development**(12)
16. Was the **intervention development** informed by the theory?
    **Yes**, if the intervention is explicitly based on a theory, predictor, combination of theories, or predictors.
    **No**, if the author explicitly stated that the intervention was not based on any theories,
    **Unclear**, if neither Yes/No.
17. If yes, describe **how the theory was used in developing the intervention**?
    As described by the author, e.g. - modelling of health behaviours.
18. Was the **theory/ predictors used to tailor intervention techniques** to recipients?
    **Yes**, if the intervention differs for different sub- groups that vary on a psychological construct (e.g., stage of change) or predictor at baseline.

**No**, if there was no tailoring of interventions
 **Unclear,** if the above is not reported in the article.

1. If yes, describe **how theory was used to tailor intervention techniques**?
   As described by the author, e.g.- Personalised feedback and interaction with the computer would promote greater self efficacy and movement in stages of change, leading to behaviour changes.
2. **Mention the theoretical constructs** (12) (modifying factors, e.g. attitudes, self-efficacy, norms) that were described by the author: They are the key concepts in the theory that are hypothesised to be causally related to behaviour and are therefore appropriate targets for the intervention. Evidence that the psychological construct relates to (correlates/predicts/causes) behaviour should be presented within the introduction or method (rather than the Discussion).
3. **Measurement of the constructs** (12)(specify how many were measured and how?): Specify any scales or questionnaires used to measure the construct of theory in relation to the intervention, for example, Self-efficacy scales.
4. Were **the mediation effects of the constructs** measured? (If so how many and how?): “mediators” are defined as ‘causal or intermediate variables that can directly affect outcomes’ or mechanisms through which an intervention might achieve its effects. For example, treatment modality, patient involvement in treatment, self-efficacy, attitudes, necessary to eat healthier, self-rated dietary habits.
5. **Theory / theories of behaviour change** **used**: Models/theories that specify relations among variables, in order to *explain* or *predict* behaviour (e.g., TPB, SCT, HBM) (13). Enter theories used for both intervention and control groups, as described by the author.

1. Attribution theory
2. Cognitive adaptation theory
3. Cognitive behavioural theory
4. Decision making theories (e.g. social judgment theory,’ fast and frugal’’ model, systematic versus heuristic decision making)
5. Decision making theory
6. Demand-control model
7. Diffusion theory
8. Effort-reward imbalance
9. Elaboration likelihood model
10. Fear arousal theory
11. Goal theories
12. Goal theory
13. Group theory (e.g. group minority theory)
14. Health beliefs model
15. Implementation theory/automotive model
16. Intrinsic motivation theories
17. Locus of control theories
18. Operant learning theory
19. Person situation contingency models
20. Precaution adoption process model
21. Relapse prevention theory
22. Self-determination theory
23. Self-regulation theory
24. Social cognitive theory
25. Social comparison theory
26. Social identity theory
27. Social identity theory
28. Social influence
29. Social learning theory
30. Social-ecological theory
31. Theory of Planned Behaviour
32. Transtheoretical model
33. Volitional control theory
34. Describe the **behaviour change techniques** used (14): They are the Strategies used to change behaviour, theory-relevant construct or predictor.
35. **Action planning:** Involves detailed planning of what the person will do including, as a minimum, when, and where to act. “When” may describe frequency (such as how many times a day/week or duration (e.g., for how long for). The exact content of action plans may or may not be described, in this case code as this technique if it is stated that the three dimensions are attended to in the action plan even if exact details are not present **NB** The terms “goal setting” or “action plan” are not enough to ensure inclusion of this technique, instances of this should be regarded as applications of technique 5 and 6.
    Action planning could include identification of sub-goals or preparatory behaviours and/or specific contexts in which the behaviour will be performed. The behaviour in this technique will be directly related to the target behaviour (e.g. shopping for healthy eating). NB – any techniques applied to preparatory behaviours should also be coded as instances of technique 9 (set graded tasks).
36. **Agree behavioural contract:** Must involve written agreement on the performance of an explicitly specified behaviour so that there is a written record of the person’s resolution witnessed by another.
37. **Barrier identification/Problem solving:** This presumes making an initial plan to change behaviour. The person is prompted to think about potential barriers and identify ways of overcoming them. Barriers may include competing goals in specified situations. This may be described as “problem solving”. If it is problem solving in relation to the performance of a behaviour, then it counts as an instance of this technique. Examples of barriers may include behavioural, cognitive, emotional, environmental, and/ or physical barriers. **NB** Closely related to techniques 7 and 9 but involves a focus on specific obstacles to performance. It contrasts with 35 which is about maintaining behaviour that has already been changed.
38. **Emotional control training:** Techniques designed to reduce negative emotions or control mood or feelings that may interfere with performance of the behaviour, and/ or to increase positive emotions that might help with the performance of the behaviour. NB Check whether there are any instances of technique 8 also, which includes identifying emotional barriers to performance, in contrast to the current technique, which addresses emotions, whether they have been identified as barriers or not.
39. **Environmental restructuring:** The person is prompted to alter the environment in ways so that it is more **supportive** of the target behaviour e.g. altering cues or reinforces. For example they might be asked to destroy all their cigarettes or all their high calorie snacks, or take their running shoes to work**.**
40. **Facilitate social comparison:** Involves explicitly drawing attention to others’ performance to elicit comparisons. **NB** the fact the intervention takes place in a group setting, or have been placed in groups on the basis of shared characteristics, does not necessarily mean social comparison is actually taking place. Social support may also be encouraged in such settings and this would then involve technique 29. Group classes may also involve instruction (technique 21) demonstration (technique 22) and practice (technique 26). Check for these additional techniques.
41. **Fear Arousal:** Involves presentation of risk and/or mortality information relevant to the behaviour with emotive images designed to evoke a fearful response (e.g., “smoking kills!” or images of the grim reaper). Do not also code instances of this technique as the more generic providing information on consequences (techniques 1 and 2).
42. **General communication skills training:** This includes any technique directed at general communication skills but not directed towards a particular behaviour change. Often this may include role play and group work focusing on listening skills or assertive skills. **NB** Practicing a particular behaviour-specific interpersonal negotiation e.g., refusal skills in relation to cigarettes or alcohol would not be an instance of this technique.
43. **Goal setting (behaviour):** The person is encouraged to make a behavioural resolution (e.g. take more exercise next week). This is directed towards encouraging people to decide to change or maintain change. **NB** This is distinguished from technique 6 and 7 as it does not involve **planning** exactly how the behaviour will be done and either when or where the behaviour or action sequence will be performed. Where the text only states that goal setting was used without specifying the detail of action planning involved then this would be an example of this technique (not technique 7).
44. **Goal setting (outcome):** The person is encouraged to set a general goal that can be achieved by behavioural means but is not defined in terms of behaviour (e.g. to reduce blood pressure or lose/maintain weight), as opposed to a goal based on changing behaviour as such. The goal may be an expected consequence of one or more behaviours, but is not a behaviour per se (see also techniques 5 and 7). This technique may co-occur with technique 5 if goals for both behaviour and other outcomes are set.
45. **Model/ Demonstrate the behaviour:** Involves ***showing*** the person how to correctly perform a behaviour e.g., through physical or visual demonstrations of behavioural performance, in person or remotely**. NB** This is distinct from just providing instruction (technique 21) because in “demonstration” the person is able to *observe* the behaviour being enacted. Techniques 21 and 22 may be used separately or together – check for this.
46. **Motivational interviewing:** This is a specific set of techniques involving prompting the person to provide self-motivating statements and evaluations of own behaviour to minimise resistance to change (includes motivational counselling). **NB** Normally this technique will be mentioned by name. Only rate this technique if explicitly referred to by name, not if one identifies specific elements of it, this may happen if you have prior experience with this technique.
47. **Plan social support/ social change:** Involves prompting the person to plan how to elicit social support from other people to help him/ her achieve their target behaviour. This will include support during interventions e.g., setting up a “buddy” system or other forms of support and following the intervention including support provided by the individuals delivering the intervention, partner, friends, family.
48. **Prompt anticipated regret:** Involves inducing expectations of future regret about the performance or non-performance of behaviour. This includes focusing on how the person will *feel* in the future and specifically whether they will feel regret or feel sorry that they did or did not take a different course of action. Do not also code instances of this technique as the more generic providing information on consequences (techniques 1 and 2).
49. **Prompt identification as role model/ position advocate:** Involves focusing on how the person may be an example to others and affect their behaviour e.g., being a good example to children. Also includes providing opportunities for participants to persuade others of the importance of adopting/ changing the behaviour. For example, giving a talk, running a peer-led session.
50. **Prompt practice:** Prompt the person to rehearse and repeat the behaviour or preparatory behaviours numerous times. Note this will also include parts of the behaviour e.g., refusal skills in relation to quitting smoking. This could be described as “building habits or routines” but is still practice so long as the person is prompted to try the behaviour (or parts of it) during the intervention or practice between intervention sessions, e.g. as “homework”.
51. **Prompt review of behavioural goals:** Involves a review or analysis of the extent to which previously set behavioural goals were achieved. In most cases this will follow previous goal setting and an attempt to act on those goals, followed by a revision or readjustment of goals, and/ or means to attain them. **NB** Check that any instance does not also involve techniques 5, 8, 9 or 11.
52. **Prompt review of outcome goals:** Involves a review or analysis of the extent to which previously set goals were achieved. In most cases this will follow previous goal setting and an attempt to act on those goals, followed by a revision of goals, and/ or means to attain them. **NB** Check that any instance does not also involve techniques 5, 8, 9 or 10.
53. **Prompt Self talk:** Encourage the person to use talk to themselves (aloud or silently) before and during planned behaviours to encourage and support action.
54. **Prompt self-monitoring of behaviour:** The person is asked to keep a record of specified behaviour/s as a method for changing behaviour. This should be an explicitly stated intervention component, as opposed to occurring as part of completing measures for research purposes. This could e.g., take the form of a diary or completing a questionnaire about their behaviour.
55. **Prompt self-monitoring of behavioural outcome:** The person is asked to keep a record of specified measures expected to be influenced by the behaviour change, e.g. blood pressure, blood glucose, weight loss, physical fitness. NB It must be reported as part of the intervention, rather than only as an outcome measure.
56. **Prompt use of imagery:** Teach the person to imagine successfully performing the behaviour or to imagine finding it easy to perform the behaviour, including component or easy versions of the behaviour. Distinct from recalling instances of previous success without imagery (technique 18).
57. **Prompting focus on past success:** Involves instructing the person to think about or list previous successes in performing the behaviour (or parts of it) before the intervention. **NB** this is not just encouragement but a clear focus on the person’s past behaviour. It is also not feedback because it refers to behaviour preceded the intervention.
58. **Prompting generalisation of a target behaviour:** Once a behaviour is performed in a particular situation, the person is encouraged or helped to try it in another situation. The idea is to ensure that the behaviour is not tied to one situation but becomes a more integrated part of the person’s life that can be performed at a variety of different times and in a variety of contexts.
59. **Provide feedback on performance:** This involves providing the participant with data about their own recorded behaviour (e.g., following technique 16) or commenting on a person’s behavioural performance (e.g., identifying a discrepancy with between behavioural performance and a set goal – see techniques 5 and 7 – or a discrepancy between one’s own performance in relation to others’ – note this could also involve technique 28; Audit and feedback involves this technique).
60. **Provide information about others’ approval:** Involves information about what other people think about the target person’s behaviour. It clarifies whether others will like, approve or disapprove of what the person is doing or will do. **NB** Check that any instance does not also involve techniques 1 or 2 or 4.
61. **Provide information on consequences of behaviour in general**: Information about the relationship between the behaviour and its possible or likely consequences **in the general case**, usually based on epidemiological data, and not personalised for the individual (contrast with technique 2).
62. **Provide information on consequences of behaviour to the individual:** Information about the ***benefits and costs*** of action or inaction **to the individual or tailored to a relevant group based on that individual’s** characteristics (i.e. demographics, clinical, behavioural or psychological information). This can include any costs/ benefits and not necessarily those related to health, e.g. feelings.
63. **Provide information on where and when to perform the behaviour:** Involves telling the person about when and where they might be able to perform the behaviour this e.g. tips on places and times participants can access local exercise classes. This can be in either verbal or written form. NB. Check whether there are also instances of technique 21.
64. **Provide instruction on how to perform the behaviour:** Involves ***telling*** the person ***how*** to perform a behaviour or preparatory behaviours, either verbally or in written form. Examples of instructions include; how to use gym equipment (without getting on and showing the participant), instruction on suitable clothing, and tips on how to take action *Showing* a person how to perform a behaviour without verbal instruction would be an instance of technique 22 only. **NB** Check whether there are also instances of techniques 5, 7, 8, 9, 22.
65. **Provide normative information about others’ behaviour:** Involves providing information about what other people are doing i.e., indicates that a particular behaviour or sequence of behaviours is common or uncommon amongst the population or amongst a specified group – presentation of case studies of a few others is not normative information. **NB** this concerns other people’s actions and is distinct from the provision of information about others’ approval (technique 3).
66. **Provide rewards contingent on effort or progress towards behaviour:** Involves praising or rewarding the person **for attempts** at achieving a behavioural goal. This might include efforts made towards achieving the behaviour, or progress made in preparatory steps towards the behaviour, but not merely participation in intervention. This can include self-reward **NB** Not reinforcement for performing the target behaviour itself (technique 13).
67. **Provide rewards contingent on successful behaviour:** Reinforcing successful performance of the specific target behaviour. This can include praise and encouragement as well as material rewards but the reward/ incentive must be explicitly linked to the achievement of the specific target behaviour i.e. the person receives the reward if they perform the specified behaviour but not if they do not perform the behaviour. This can include self-reward **NB** Check the distinction between this and techniques 7 and 17.
68. **Relapse prevention/ Coping planning:** This relates to planning how to maintain behaviour that has been changed. The person is prompted to identify in advance situations in which the changed behaviour may not be maintained and develop strategies to avoid or manage those situations. Contrast with techniques 7 and 8 which are about initiating behaviour change.
69. **Set graded tasks:** Breaking down the target behaviour into smaller easier to achieve tasks and enabling the person to build on small successes to achieve target behaviour. This may include increments towards a target behaviour, or incremental increases from baseline behaviour. **NB** The key difference to technique 7 lies in planning to perform a sequence of preparatory actions (e.g. remembering to take gym kit to work), task components or target behaviours which are in a logical sequence or *increase in difficulty over time* - as opposed to planning “**if-then” contingencies** when/where to perform behaviours.
70. **Shaping:** Contingent rewards are first provided for any approximation to the target behaviour e.g., for any increase in physical activity. Then, later, only a more demanding performance, e.g., brisk walking for 10 minutes on three days a week would be rewarded. Thus, this is graded use of contingent rewards over time.
71. **Stress management:** This is a set of specific techniques (e.g., progressive relaxation) which do not target the behaviour directly but seek to reduce anxiety and stress to facilitate the performance of the behaviour. **NB** Only rate this technique if explicitly referred to by name, not if one identifies specific elements of it, this may happen if you have prior experience with this technique.
72. **Teach to use prompts/ cues:** The person is taught to identify environmental prompts which can be used to **remind** them to perform the behaviour (or to perform an alternative, incompatible behaviour in the case of behaviours to be reduced). Cues could include times of day, particular contexts or elements of contexts which prompt them to perform the target behaviour. Note that this could be used independently or in conjunction with techniques 5 and 7 (see also 24).
73. **Time management:** This includes any technique designed to teach a person how to manage their time in order to make time for the behaviour. These techniques are not directed towards performance of target behaviour but rather seek to facilitate it by freeing up times when it could be performed. **NB** Only rate this technique if explicitly referred to by name, not if one identifies specific elements of it, this may happen if you have prior experience with this technique.
74. **Use of follow up prompts:** Intervention components are gradually reduced in intensity, duration and frequency over time, e.g. letters or telephone calls instead of face to face and/or provided at longer time intervals
75. **OUTCOME MEASURES**
76. **Dietary assessment (Primary outcomes)**
77. **Data Collection Methods**

**Self-administered**: The participant completes a questionnaire by self without any involvement of the interviewer.

**Interviewer-administered**: An interviewer either conducts or assists at an interview.

1. **Data collection modes**

**Postal**: The participant receives and returns a questionnaire by post.

**Telephone**: The interviewer telephones the participant to collect the information.

**Computer**: Participant completes a questionnaire on a computer.

**Face-to-face**: Interviewer fills in a questionnaire during interview.

1. **Diet Assessment Tools/Techniques Used** (15-16) As well as indicating the method used (as reported), also state whether any validation of the dietary assessment method used was reported. (Validity describes the degree to which a dietary method measures what it is intended to measure.) If yes, then describe the nutrients for which the validity was assessed.
2. **Estimated food records**
   The respondent is asked to record, at the time of consumption, all foods and beverages (including snacks) eaten in household measures, for a specified time period. It will also record detailed descriptions of all foods and beverages (including brand names).
3. **Weighed food records**
   The participant is instructed to weigh all food and beverages consumed during a specified time period, and record amounts eaten. Details of methods of food preparation, description of foods, and brand names (if known) are also recorded.
4. **24-Hour Recall** The interviewer asks the participants to recall their exact food intake during the previous 24-h period or preceding day, usually with protion estimates, probed by the interviewer.
5. **Diet History**
   Attempts to estimate the usual food intake and meal pattern of individuals over a relatively longer period of time – often a month.
6. **Food Frequency Questionnaire (FFQ)**FFQ aims to assess the frequency with which food items or food groups are consumed during a specified time period. The questionnaire consists of a list of foods and an associated set of frequency-of-use response categories, often plus portion sizes.
   **Time period**: mention here the frequency-of-use response categories that may be daily, weekly, monthly, or yearly.
7. **Clinical assessment (Secondary outcomes)**
8. **Data Collection Methods**

**Self-administered**: The participant completes a questionnaire by self without any involvement of an interviewer.

**Interviewer-administered**: An interviewer either conducts or assists at an interview.

**Objectively measured**: Instruments are used to collect data, e.g. scales; callipers; biochemical tests.

1. **Data collection modes**

**Postal**: The participant receives and returns a questionnaires by post.

**Telephone**: The interviewer telephones the participant to collect the information.

**Computer**: Participant completes a questionnaire on a computer.

**Face-to-face**: Interviewer fills in a questionnaire during an interview.

1. **Clinical Assessment Tools/Techniques Used**
2. **Anthropometric techniques**

Specify any anthropometric tools or techniques used (e.g. weighing, BMI, skin fold thickness, waist circumference), and note whether any validation of the technique(s) is reported.

1. **Biochemical techniques**

Specify any biochemical tools or techniques used (e.g. ELISA, Hemocue), and note whether any validation of the technique(s) is reported.

1. **Primary Outcomes**

Provide effect size information (mean, standard deviation, statistic type, value of statistic, *p*-value, direction of effect), number of responders if they are available). Note outcomes at baseline first, then at follow-up. **Tables may have to be modified according to data collection intervals** (e.g. If there are only two groups and outcomes were collected at two periods, e.g. at 3m & 6m, then use the 1^st^ column for the results for 3m & the 2^nd^ for the 6m. Add an extra column for the comparator.)

1. **Energy & Nutrients**

For each nutrient or energy, note the mean / median intake in each intervention group and the comparison (control) group as reported.

1. **Prevalence Rates for Energy & Nutrients**

For each nutrient or energy, note the prevalence of adequate / inadequate intake, as reported in the study, as well as the criteria used to assess this (Eg. .% of energy / portions per day / grams per day etc).

1. **Food Group Outcomes**

For each food group, note the unit of measurement (Eg. .% of energy / portions per day / grams per day etc), and note the mean and proportion consuming the food group in each group, as reported.

1. **Secondary Outcomes**

Provide effect size information (mean, standard deviation, statistic type, value of statistic, *p*-value, direction of effect), number of responders if they are available). Note outcomes at baseline first, then at follow-up. **Tables may have to be modified according to data collection intervals** (e.g. If there are only two groups and outcomes were collected at two periods, e.g. at 3m & 6m, then use the 1^st^ column for the results for 3m & the 2^nd^ for the 6m. Add an extra column for the comparator.)

- - 1. **Anthropometric / biochemical**

Note the mean / median for each indicator reported in each group, as well as the tool / method used to assess the indicator (Eg. Waist measurements, DEXA, HemoCue haemoglobin test, etc)

- - 1. **Prevalence Rates for Secondary Outcomes**

For each indicator reported, note the prevalence (%) in each group, plus the criteria used (Eg. Overweight = BMI 25-30 kg/m2)

1. **Mechanisms of change**

Report on mediators (cognitive and emotional), antecedents, consequences, and organisation or environmental influences only when primary or secondary outcomes are reported. Under ‘tools used’, name the questionnaire used to collect the data.

1. **Additional measures of engagement**

Report results of other measures of compliance reported in the trial.

1. **Further comments**

Other useful information about the trial, e.g. results of a process evaluation.

1. Specify whether **the above findings were explained in relation to theory, or theories**, on which the interventions was based? (12)

Report, if noted in the discussion, whether the authors confirmed their findings in relation to the theory/theories.

**REFERENCES**

1. Tworoger SS, Yasui Y, Ulrich CM, Nakamura H, LaCroix K, Johnston R, et al. Mailing Strategies and Recruitment into an Intervention Trial of the Exercise Effect on Breast Cancer Biomarkers. Cancer Epidemiology, Biomarkers & Prevention. 2002;11:73-7.

2. Smith AD, Manna DR. E-recruitment of patients for clinical trials. International Journal of Electronic Healthcare 2005;1(4):413-26.

3. Wang D, Nitsch D, Bakhai A. Randomized Clinical Trials. In: Wang D, Bakhai A, editors. Clinical Trials: A Practical Guide to Design, Analysis, and Reporting. London: Remedica; 2006.

4. Clayton T. Fundamentals of Clinical Trials: Randomisation. London: London School of Hygiene & Tropical Medicine; 2009.

5. Wang D, Bakhai A. Randomization. In: Wang D, Bakhai A, editors. Clinical Trials: A Practical Guide to Design, Analysis, and Reporting. London: Remedica; 2006.

6. Moher D, Schulz KF, Altman DG, for the CONSORT Group. The CONSORT statement: revised recommendations for improving the quality of reports of parallel-group randomised trials. Lancet. 2001;357:1191–94.

7. Clemens F, Elbourne D, Merle C, Edwards S, Kirwan B. Clinical Trials in Practice: Recruitment and Randomisation. London: London School of Hygiene & Tropical Medicine; 2006.

8. Davidson KW, Goldstein M, Kaplan RM, Kaufmann PG, Knatterud GL, Orleans CT, et al. Evidence-Based Behavioral Medicine: What Is It and How Do We Achieve It? Annals of Behavioral Medicine. 2003;26(3):161-71.

9. Higgins JPT, Green S, editors. Cochrane Handbook for Systematic Reviews of Interventions2009.

10. Thomas BH, Ciliska D, Dobbins M, Micucci S. Quality Assessment Tool for Quantitative Studies: Effective Public Health Practice Project, McMaster University; 2009.

11. Thomas BH, Ciliska D, Dobbins M, Micucci S. Quality Assessment Tool for Quantitative Studies Dictionary: The Effective Public Health Practice Project (EPHPP), McMaster University; 2008.

12. Michie S, Prestwich A. Are interventions theory-based? Development of a Theory Coding Scheme Health Psychology. 2010;29(1):1-8.

13. Michie S, Johnston M, Abraham C, Lawton R, Parker D, Walker A, et al. Making psychological theory useful for implementing evidence based practice: a consensus approach. Quality and Safety in Health Care. 2005;14:26–33.

14. Abraham C, Michie S. A Taxonomy of Behavior Change Techniques Used in Interventions. Health Psychology. 2008;27(3):379–87.

15. Bingham S. Dietary assessment. In: Mann J, Truswell AS, editors. Essentials of human nutrition

Oxford: Oxford University Press; 2007.

16. Gibson RS. Principles of nutritional assessmnet. 2nd ed. New York: Oxford University Press; 2005.
